# The burden of chronic kidney disease attributable to diet low in whole grains from 1990 to 2021: a global, regional and national analysis

**DOI:** 10.3389/fnut.2026.1684886

**Published:** 2026-02-20

**Authors:** Zuzhi Zhao, Peng Sun, Zhengshi Xia, Suqin Zhang, Mengfei Xu, Jianhua Li, Pengfei Xu

**Affiliations:** 1 Department of Thyroid Surgery, The First Affiliated Hospital of Zhengzhou University, Zhengzhou, China; 2 The Children's Hospital, The First Affiliated Hospital of Zhengzhou University, Zhengzhou, China; 3 Clinical Systems Biology Laboratories, Translational Medicine Center, The First Affiliated Hospital of Zhengzhou University, Zhengzhou, China

**Keywords:** chronic kidney disease, death, diet low in whole grains, disability-adjusted life years, global burden of disease

## Abstract

**Background:**

Chronic kidney disease (CKD) is a major global health issue with a growing disease burden. Dietary factors play a significant role in the onset and progression of CKD. Currently, there is a lack of comprehensive assessment on the global CKD burden attributable to diet low in whole grains, highlighting an urgent need to analyze its cross-regional and temporal distribution characteristics and trends based on standardized metrics.

**Methods:**

Using Global Burden of Disease (GBD) 2021 data, we analyzed CKD deaths, disability-adjusted life years (DALYs), age-standardized mortality rates (ASMR), and age-standardized DALY rates (ASDR) attributable to low whole-grain intake across 204 countries (1990–2021). Temporal trends were quantified via estimated annual percentage change (EAPC); autoregressive integrated moving average (ARIMA) modeling projected burdens to 2050.

**Results:**

In 2021, global deaths and DALYs from CKD attributable to diet low in whole grains were 21,992.9 and 549,741, respectively, increasing by 183 and 146% compared with 1990. Both the ASMR and ASDR showed upward trends. At the regional level, in 2021, the middle SDI region had the highest deaths and DALYs, while the low SDI region had the highest ASMR and ASDR; the East Asia region carried the heaviest burden overall. At the national level, China ranked first in both the number of deaths and DALYs, whereas American Samoa had the highest ASMR and ASDR. In 2021, the EAPC of ASMR for CKD due to diet low in whole grains was negatively correlated with the SDI (*R* = −0.517, *p* < 0.001), as did ASDR. ARIMA predicted rising deaths, DALYs, and ASMR by 2050. ASDR will stabilize overall but rise in women.

**Conclusion:**

Over the past three decades, the global burden of CKD attributable to diet low in whole grains has increased and remains at a high level, with significant regional disparities. Policymakers must implement targeted measures to enhance public awareness and intake of whole grains.

## Introduction

Chronic kidney disease (CKD) represents a critical global public health challenge, with escalating prevalence and economic burden driven by demographic transitions and modifiable risk factors (1). Dietary patterns significantly influence CKD pathogenesis, yet the quantifiable impact of specific dietary components remains incompletely characterized (2). Whole grains—rich sources of dietary fiber, magnesium, and antioxidants—exert protective effects against metabolic dysregulation, inflammation, and oxidative stress, mechanisms implicated in CKD progression (3, 4). Substantial evidence links higher whole-grain intake to reduced risks of diabetes and hypertension, key CKD etiologies (5). Conversely, a diet low in whole grains may exacerbate renal impairment through these pathways.

Despite established biological plausibility, the global disease burden of CKD attributable to diet low in whole grains lacks comprehensive assessment. Prior studies have primarily focused on individual nutrient effects or regional cohorts, leaving a gap in population-level quantification of this modifiable risk factor (6–8). The Global Burden of Disease (GBD) framework provides standardized methodologies to estimate attributable burdens across geographies and demographics, enabling evidence-based prioritization (9). Critically, no study has systematically analyzed spatiotemporal trends, sociodemographic disparities, or future projections of CKD burdens specifically tied to diet low in whole grains.

This study leverages GBD 2021 data to address these gaps. We quantify the deaths and DALYs attributable to diet low in whole grains across 204 countries and territories from 1990 to 2021. Using age-standardized rates (ASRs), socio-demographic index (SDI) stratification, and regression analyses, we characterize cross-national inequalities and temporal trajectories. Furthermore, autoregressive integrated moving average (ARIMA) modeling projects burden trends through 2050. Our findings aim to inform targeted nutritional interventions and health policies to mitigate the growing CKD epidemic.

## Materials and methods

### Data resources and collection

All data were obtained from the 2021 GBD Study database.^1^ GBD is a publicly accessible repository providing estimates for the burden of 371 diseases and injuries across 204 countries and territories from 1990 to 2021, including incidence, prevalence, mortality, disability-adjusted life years (DALYs), years lived with disability (YLDs), years of life lost (YLL), and healthy life expectancy (HALE) (10). From GBD 2021, we extracted data on CKD deaths, DALYs, age-standardized mortality rate (ASMR), and age-standardized DALY rate (ASDR) attributable to diet low in whole grains. Based on the extracted data, we characterized the CKD burden attributable to diet low in whole grains by year, sex, 5-year age bands from 25 to 94 years and ≥95 years, SDI regions, and countries.

### Definitions

Whole grain intake was defined as the average daily consumption (in grams) of whole grains and their products, encompassing breakfast cereals, bread, rice, biscuits, muffins, tortillas, pancakes, pasta, and other relevant sources. Due to variations in dietary patterns across different regions, international organizations such as the World Health Organization (WHO) and the Food and Agriculture Organization (FAO) have not established a globally uniform absolute value to define adequate whole grain intake. Nevertheless, they explicitly recommend sufficient daily consumption of whole grains. Various countries and regions have set their own recommended daily intake levels for whole grains, tailored to local dietary habits, generally ranging from 50 g to 180 g per day (11–13). In this study, insufficient intake of whole grains is defined as a population’s daily consumption falling below the region-specific Theoretical Minimum Risk Exposure Level (TMREL) for whole grains. Specifically, the methodology involves synthesizing data from multiple sources—including dietary recall surveys, food frequency questionnaires, household budget surveys, and food supply data from the FAO—to estimate the population mean daily intake of whole grains (typically in grams per day), stratified by region, country, age, and sex. This estimated intake is then compared against the TMREL, which is determined based on the optimal intake level associated with the lowest risk of disease, as derived from the best available scientific evidence, such as meta-analyses of prospective cohort studies. The extent to which the actual intake falls below the TMREL is quantified as the risk exposure level, which subsequently serves as the basis for calculating the attributable burden of disease.

DALY is a metric quantifying the total health loss within a population due to morbidity, disability, and premature mortality, combining YLL with YLD. The ASR represents a rate adjusted to a standard population age structure. As crude mortality and DALY rates are influenced both by age-specific rates and the population’s age distribution, the ASR enables the removal of age-structure effects, permitting more accurate comparisons of overall mortality and DALYs burdens across regions or time periods.

### Socio-demographic index (SDI)

The SDI, a composite indicator of socio-demographic development, is derived from an extensive assessment including total fertility rate among females under age 25, average educational attainment among individuals aged ≥15 years, and per capita income distribution, scaled from 0 to 1 (10). It demonstrates a strong correlation with health-related outcomes and measures of disease burden, rendering it a valuable tool for assessing health development status and disease burden across countries and regions. Based on SDI values, all countries and territories were categorized into five quintiles: low SDI, low-middle SDI, middle SDI, high-middle SDI, and high SDI.

### Statistical analyses

The estimated annual percentage change (EAPC) is a widely used metric for quantifying trends in age-standardized rates over specific periods (14). In this study, we computed the EAPC and its 95% confidence interval (95% CI) to assess temporal trends in the ASMR and ASDR for CKD attributable to diet low in whole grains from 1990 to 2021. The ASR per 100,000 population was calculated using the following formula:


$$\mathrm{ASR}=\frac{\sum_{i=1}^{A}\;a_{i}w_{i}}{\sum_{i=1}^{A}\;w_{i}}\times 100,000$$

Where *α* i: the age-specific rate in ith the age group; w: the number of people in the corresponding ith age group among the standard population; A: the number of age groups.

We constructed a linear regression model: y = α + *β*x + *ε* where x represents the calendar year, and y denotes the natural logarithm of either ln(ASMR) or ln(ASDR). The EAPC and its 95% confidence interval (CI) were derived using the formula: 100 × (exp(β) − 1) (15, 16). A consistent increasing trend in ASMR or ASDR was concluded when both the EAPC point estimate and its lower 95% CI boundary exceeded zero. Conversely, a decreasing trend was established when both values were below zero. Instances where the 95% CI overlapped zero indicated temporal stability. Spearman correlation analysis evaluated associations between SDI and ASMR or ASDR values.

We employed the Concentration Index (CI) and the Slope Index of Inequality (SII) to quantify the socioeconomic inequality in the kidney disease burden attributable to low whole grain intake across quintiles of the SDI. The CI was calculated based on individuals or population subgroups ranked by SDI, with the ranking variable being the SDI quintiles, and its value ranges from −1 to 1. In our study, a positive value indicates that the disease burden is more concentrated in regions with higher SDI, whereas a negative value signifies a greater concentration of the burden in lower-SDI regions. A larger absolute value reflects a higher degree of inequality. The SII, in contrast, captures the absolute difference in disease burden by representing the disparity between the highest and lowest SDI groups.

Future disease burden trajectories were projected using ARIMA models (17). ARIMA is optimal for time-series data exhibiting linear trends and low volatility, effectively capturing intrinsic autocorrelations in historically driven stable patterns. This approach assumes: data stationarity after differencing, linear fluctuations without external nonlinear shocks or complex seasonality and future values depend solely on historical observations and error terms.

Differencing was applied to achieve stationarity before model specification. Optimal combinations of autoregressive (p), differencing (d), and moving average (q) orders were determined separately for subgroups stratified by SDI level and sex. Model selection prioritized the lowest Akaike Information Criterion (AIC) score, which balances goodness-of-fit against model complexity through parameter count penalties to mitigate overfitting. Among competing models, those achieving minimal AIC were further evaluated. Final model optimization required: high explanatory power measured by *R*^2^ and low prediction error quantified by root mean square error (RMSE).

Models satisfying minimal AIC with maximal *R*^2^ and minimal RMSE were selected as optimal due to superior fit and predictive accuracy. Residual diagnostics via Ljung-Box testing confirmed white noise properties to validate model adequacy. The conditional forecast variance for each future prediction point (with forecast horizon *h*) is computed via a recursive algorithm, utilizing the residual variance estimated by the model and the covariance matrix of the model parameters. For an ARIMA model, the forecast variance Var (e _*t* + *h*_) for the prediction at time *t* + *h* increases with the forecast horizon *h*. This calculation incorporates the uncertainty associated with the estimation of the model parameters and the cumulative impact of future random shocks. The prediction intervals are then derived under the assumption of a normal distribution, given by the formula:


$${\hat{y\;}}_{t+h}\pm Z_{\alpha /2}.\sqrt{\mathrm{Var}(e_{t+h})}$$

Where ${\hat{y\;}}_{t+h}$ is the point forecast at horizon *h*, $Z_{\alpha /2}$ is the critical value from the standard normal distribution corresponding to the desired confidence level 1 − *α*, and $\sqrt{\mathrm{Var}(e_{t+h})}$ is the standard deviation of the forecast error.

Statistical analysis and visualization were implemented in R software (v4.4.2).

## Results

### Global level

**Figure 1:**
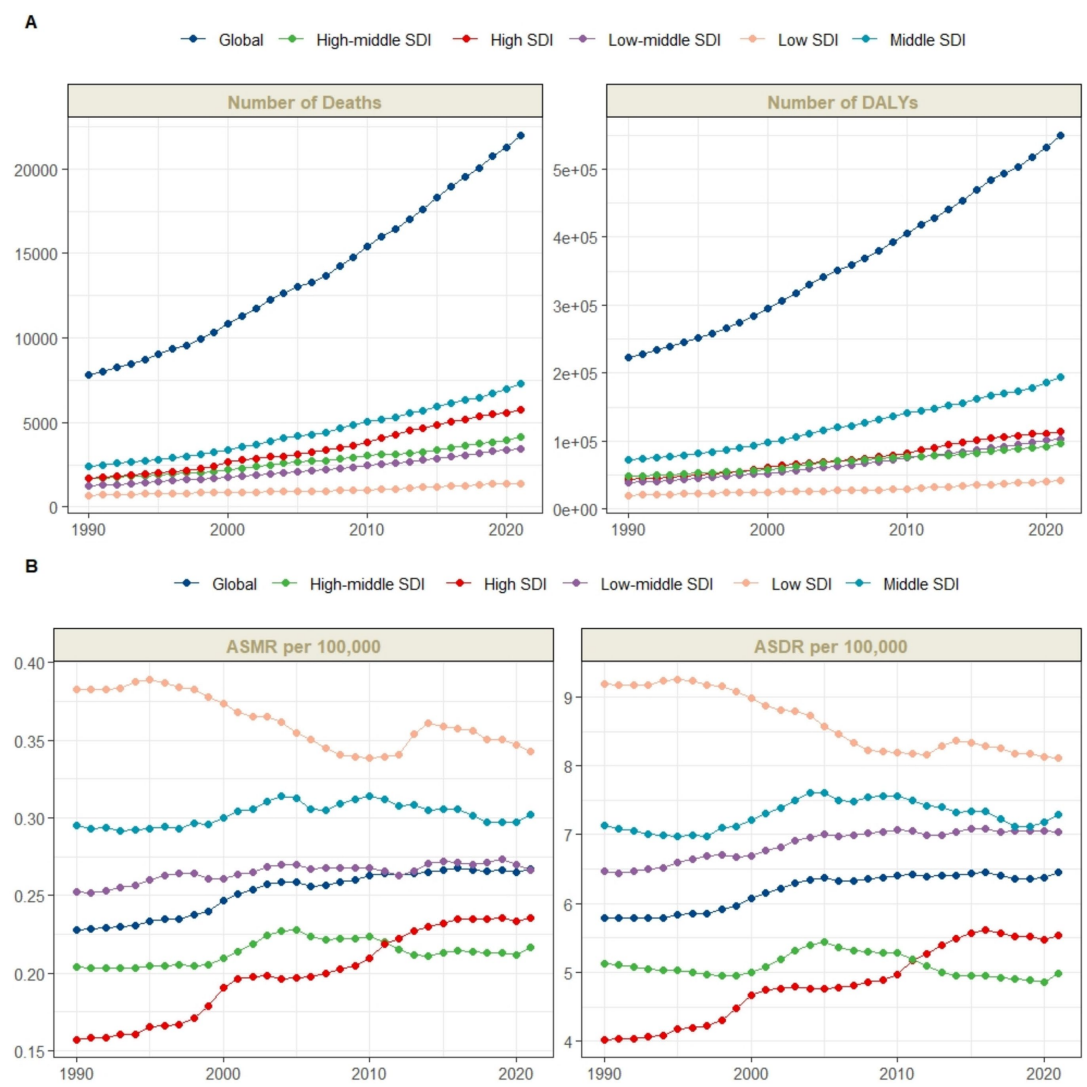
The CKD burden attributable to diet low in whole grains by global and SDI region. **(A)** The number of CKD deaths and DALYs attributable to diet low in whole grains from 1990 to 2021; **(B)** The age-standardized rate of CKD deaths and DALYs attributable to diet low in whole grains from 1990 to 2021. DALYs, Disability-adjusted life years; ASMR, Age-standardized mortality rate; ASDR, Age-standardized DALY rate.

**Figure 2:**
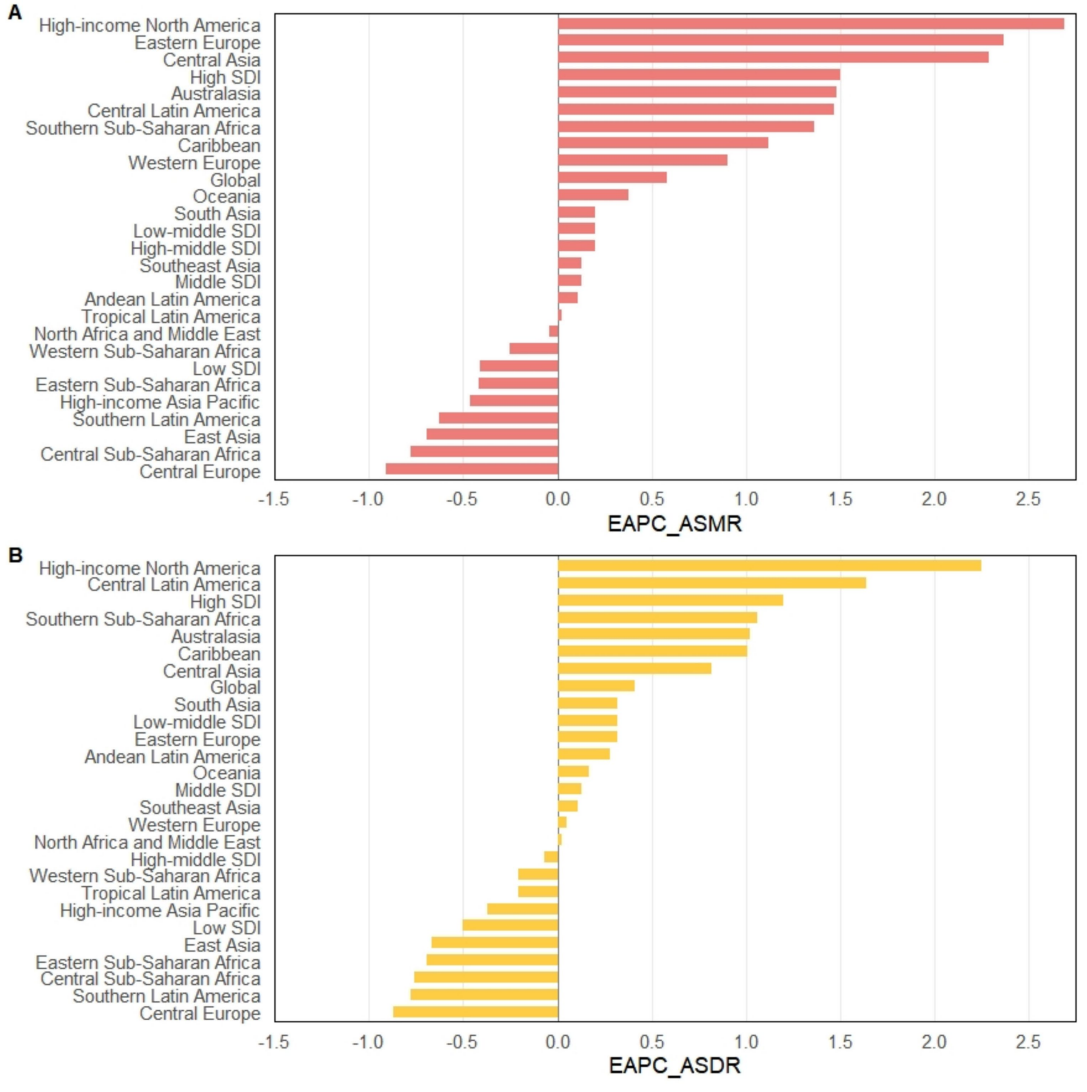
EAPC trends in ASMR **(A)** and ASDR **(B)** of CKD attributable to diet low in whole grains by global, SDI regions, and GBD regions from 1990 to 2021. ASMR, Age-standardized mortality rate; ASDR, Age-standardized DALY rate; EAPC, Estimated annual percentage changes.

From 1990 to 2021, the global number of deaths from CKD attributable to diet low in whole grains increased from 7,782.5 (95% UI: 2,000.9 to 14,295.7) to 21,992.9 (95% UI: 5,440.5 to 41,242.4), representing a 183% increase. Similarly, the number of DALYs rose from 223,309.3 (95% UI: 55,683.3 to 421,922.3) to 549,741 (95% UI: 135,764.8 to 1,065,976.2), an increase of 146%. Furthermore, the ASMR increased from 0.23 (95% UI: 0.06 to 0.41) to 0.27 (95% UI: 0.07 to 0.50) per 100,000 population, with an EAPC of 0.58 (95% CI: 0.51 to 0.65). Concurrently, the ASDR rose from 5.80 (95% UI: 1.44 to 10.99) to 6.46 (95% UI: 1.59 to 12.52) per 100,000, with an EAPC of 0.41 (95% CI: 0.34 to 0.47) (Table 1; Figures 1, 2).

**Table 1:** Burden of CKD attributable to diet low in whole grains in Global, SDI regions, and GBD regions, 1990–2021.

location	1990		2021		EAPC_95%CI
	Number (95% UI)	ASR (95% UI)	Number (95% UI)	ASR (95% UI)	
Deaths					
Global	7782.5 (2000.9–14295.7)	0.23 (0.06–0.41)	21992.9 (5440.5–41242.4)	0.27 (0.07–0.5)	0.58 (0.51 to 0.65)
SDI regions					
High SDI	1715.6 (436.9–3275.8)	0.16 (0.04–0.3)	5744.9 (1445.4–10809.7)	0.24 (0.06–0.44)	1.5 (1.39 to 1.61)
High-middle SDI	1680.7 (425.6–3104.3)	0.2 (0.05–0.38)	4129.6 (1046.2–7789.1)	0.22 (0.05–0.41)	0.2 (0.08 to 0.32)
Middle SDI	2417.3 (613.9–4367.4)	0.29 (0.07–0.53)	7284.3 (1781.9–13784.8)	0.3 (0.07–0.57)	0.13 (0.05 to 0.21)
Low-middle SDI	1,261 (320.3–2306.6)	0.25 (0.06–0.46)	3410.7 (888.9–6478.4)	0.27 (0.07–0.5)	0.2 (0.15 to 0.25)
Low SDI	698.7 (170.7–1323.1)	0.38 (0.1–0.72)	1403.5 (345.6–2,646)	0.34 (0.09–0.64)	−0.41 (−0.51 to −0.31)
GBD regions					
Andean Latin America	107.8 (27.2–197.8)	0.59 (0.15–1.09)	360.6 (90–666.1)	0.63 (0.16–1.16)	0.11 (−0.23 to 0.46)
Australasia	24.4 (5.6–47.8)	0.11 (0.03–0.22)	93.6 (22.4–181.7)	0.15 (0.04–0.29)	1.48 (1.21 to 1.74)
Caribbean	94.9 (24–162.8)	0.4 (0.1–0.69)	254.5 (65.7–448)	0.47 (0.12–0.82)	1.12 (0.92 to 1.33)
Central Asia	21.9 (4.9–44.8)	0.05 (0.01–0.1)	87.3 (19–175.4)	0.11 (0.03–0.23)	2.29 (1.78 to 2.79)
Central Europe	226.8 (56.5–442.7)	0.16 (0.04–0.32)	293.1 (70.4–552.1)	0.13 (0.03–0.24)	−0.91 (−1.11 to −0.71)
Central Latin America	258.2 (64.5–480.1)	0.36 (0.09–0.66)	1,191 (308.5–2208.7)	0.48 (0.12–0.9)	1.47 (0.99 to 1.96)
Central Sub-Saharan Africa	124.5 (31.7–242.1)	0.72 (0.19–1.38)	266.8 (69.6–556.2)	0.61 (0.17–1.26)	−0.78 (−0.9 to −0.66)
East Asia	1943.2 (493.6–3569.9)	0.31 (0.08–0.56)	4999.3 (1282.9–9456.7)	0.26 (0.07–0.49)	−0.69 (−0.82 to −0.56)
Eastern Europe	76 (17.6–159.7)	0.03 (0.01–0.06)	217.4 (54.4–432)	0.06 (0.01–0.12)	2.37 (1.98 to 2.75)
Eastern Sub-Saharan Africa	277.1 (65.8–536.1)	0.46 (0.11–0.88)	550.2 (128.6–1063.2)	0.44 (0.1–0.85)	−0.42 (−0.5 to −0.34)
High-income Asia Pacific	382.5 (91.7–729)	0.23 (0.05–0.43)	1314.9 (345.1–2502.3)	0.19 (0.05–0.36)	−0.46 (−0.59 to −0.32)
High-income North America	579.9 (156–1110.4)	0.16 (0.04–0.3)	2460.7 (642.9–4518.5)	0.35 (0.09–0.65)	2.69 (2.49 to 2.9)
North Africa and Middle East	669.4 (163.6–1286.4)	0.5 (0.12–1)	1898.6 (485.8–3615.7)	0.49 (0.12–0.95)	−0.04 (−0.18 to 0.1)
Oceania	9.1 (2.4–17)	0.4 (0.11–0.74)	26.7 (6.9–49.2)	0.45 (0.12–0.82)	0.38 (0.3 to 0.46)
South Asia	930.8 (240–1751.2)	0.19 (0.05–0.36)	2708.2 (691.7–5218.6)	0.2 (0.05–0.39)	0.2 (0.11 to 0.3)
Southeast Asia	452.3 (112.3–852.4)	0.21 (0.05–0.41)	1290.8 (324.3–2469.4)	0.22 (0.06–0.43)	0.13 (0.1 to 0.15)
Southern Latin America	230.8 (58.5–437.3)	0.54 (0.14–1.02)	366.9 (96.6–698.3)	0.4 (0.11–0.77)	−0.63 (−1.01 to −0.25)
Southern Sub-Saharan Africa	45.6 (10.5–87.9)	0.19 (0.05–0.37)	136.9 (34.3–280.2)	0.27 (0.07–0.55)	1.36 (1.06 to 1.67)
Tropical Latin America	281.7 (68.7–511.6)	0.36 (0.09–0.66)	916.9 (235.8–1697.9)	0.37 (0.09–0.68)	0.02 (−0.14 to 0.19)
Western Europe	773.2 (198–1545.9)	0.13 (0.03–0.26)	1967.9 (490.9–3854.4)	0.15 (0.04–0.29)	0.9 (0.74 to 1.06)
Western Sub-Saharan Africa	272.5 (66.6–521.2)	0.4 (0.1–0.75)	590.5 (150.4–1130.7)	0.38 (0.1–0.73)	−0.25 (−0.34 to −0.16)
DALYs					
Global	223309.3 (55683.3–421922.3)	5.8 (1.44–10.99)	549,741 (135764.8–1065976.2)	6.46 (1.59–12.52)	0.41 (0.34 to 0.47)
SDI regions					
High SDI	43820.5 (10671.9–83900.8)	4.03 (0.98–7.69)	114341.3 (28795.3–211357.1)	5.53 (1.38–10.31)	1.2 (1.11 to 1.3)
High-middle SDI	48009.8 (11809.5–93379.8)	5.13 (1.25–9.96)	96423.7 (23871.6–187357.7)	4.98 (1.23–9.78)	−0.07 (−0.19 to 0.05)
Middle SDI	72092.3 (17885.2–135,121)	7.14 (1.78–13.4)	193559.8 (47220.6–376037.2)	7.29 (1.77–14.16)	0.13 (0.04 to 0.23)
Low-middle SDI	38771.9 (9642.7–74274.3)	6.46 (1.62–12.16)	103148.6 (25565.8–202387.3)	7.03 (1.76–13.65)	0.32 (0.27 to 0.37)
Low SDI	20346.2 (4812.5–39176.8)	9.19 (2.25–17.69)	41,771 (9658.2–80100.4)	8.12 (1.96–15.45)	−0.5 (−0.57 to −0.43)
GBD regions					
Andean Latin America	2500.4 (582–4633.5)	12.52 (2.98–23.17)	8297.2 (2108.3–15387.1)	13.99 (3.55–25.69)	0.28 (−0.04 to 0.6)
Australasia	600.9 (144–1165.9)	2.62 (0.62–5.09)	1839.9 (437–3522.3)	3.27 (0.77–6.35)	1.02 (0.8 to 1.24)
Caribbean	2552.6 (642.7–4,456)	9.86 (2.47–17.24)	6145.4 (1542–11,021)	11.42 (2.87–20.49)	1.01 (0.83 to 1.18)
Central Asia	1,608 (354.7–3,293)	3.38 (0.75–6.87)	4032.5 (919.1–8,169)	4.68 (1.07–9.51)	0.82 (0.55 to 1.1)
Central Europe	7093.8 (1756.1–13832.3)	4.93 (1.22–9.62)	7839.4 (1918.3–15,070)	3.71 (0.91–7.12)	−0.87 (−0.98 to −0.77)
Central Latin America	7,746 (1939.7–14,920)	9 (2.25–16.81)	33769.8 (8697.5–63271.7)	13.13 (3.39–24.63)	1.64 (1.19 to 2.09)
Central Sub-Saharan Africa	3978.7 (965.6–7835.3)	17.44 (4.32–33.81)	8871.4 (2218.1–18455.8)	14.84 (3.84–30.49)	−0.76 (−0.87 to −0.65)
East Asia	56037.1 (13635.7–106632.6)	6.94 (1.73–13.13)	120859.4 (29793.1–239415.2)	5.7 (1.4–11.29)	−0.67 (−0.82 to −0.52)
Eastern Europe	5212.8 (1161.9–10979.1)	1.96 (0.43–4.1)	7934.7 (1913.1–16270.4)	2.3 (0.55–4.78)	0.32 (0.2 to 0.44)
Eastern Sub-Saharan Africa	6902.8 (1619.8–13219.7)	9.75 (2.31–18.78)	13242.1 (3021–25414.9)	8.54 (2–16.34)	−0.69 (−0.77 to −0.6)
High-income Asia Pacific	8402.1 (1902.2–16025.4)	4.46 (1.01–8.43)	20672.7 (5184.9–39927.4)	3.73 (0.92–7.07)	−0.37 (−0.52 to −0.21)
High-income North America	15522.3 (3921.8–30276.7)	4.48 (1.13–8.69)	52560.9 (13934.1–95223.4)	8.47 (2.26–15.23)	2.25 (2.07 to 2.43)
North Africa and Middle East	17821.4 (4322.9–34,447)	10.98 (2.69–21.39)	50850.6 (12654.1–100,658)	10.96 (2.76–21.13)	0.02 (−0.05 to 0.09)
Oceania	297.2 (72.8–543.2)	9.94 (2.54–18.11)	791.8 (204.1–1502.6)	10.59 (2.77–19.72)	0.17 (0.11 to 0.24)
South Asia	32099.8 (8070–62574.1)	5.5 (1.4–10.64)	90099.1 (21894.3–178686.8)	5.94 (1.46–11.8)	0.32 (0.26 to 0.38)
Southeast Asia	13145.8 (3130.7–25204.2)	5.27 (1.29–10.28)	35682.8 (8602.1–70211.3)	5.42 (1.34–10.61)	0.11 (0.09 to 0.12)
Southern Latin America	5134.3 (1259.7–9,637)	11.35 (2.77–21.25)	7177.5 (1770.4–13309.5)	8.21 (2.01–15.34)	−0.78 (−1.08 to −0.47)
Southern Sub-Saharan Africa	1586.8 (358.5–3107.1)	5.62 (1.29–10.76)	4,517 (1110.7–9346.3)	7.42 (1.83–15.15)	1.06 (0.81 to 1.31)
Tropical Latin America	8705.6 (2076.8–16268.8)	9.32 (2.22–17.17)	23477.3 (6033.7–43712.9)	9.12 (2.34–16.98)	−0.21 (−0.37 to −0.05)
Western Europe	18516.6 (4447–37221.4)	3.19 (0.75–6.34)	32705.5 (8006.1–63568.2)	3.06 (0.75–5.93)	0.05 (−0.03 to 0.13)
Western Sub-Saharan Africa	7844.3 (1777.4–15274.1)	9.32 (2.21–17.66)	18373.9 (4516–35913.2)	9.1 (2.31–17.17)	−0.21 (−0.29 to −0.14)

SDI, Socio-demographic Index; GBD, Global Burden of Disease study; ASMR, Age-standardized mortality rate; DALYs, Disability-adjusted life years; ASDR, Age-standardized DALY rate; EAPC, Estimated annual percentage changes; UI, Uncertainty interval; CI, confidence interval.

### Regional level

At the SDI regional level, in 2021, the middle SDI region had the highest number of CKD deaths attributable to diet low in whole grains, totaling 7,284.3 (95% UI: 1,781.9 to 13,784.8), while the low SDI region had the lowest, at 1,403.5 (95% UI: 345.6 to 2,646). Similarly, the middle SDI region bore the highest burden of CKD DALYs due to diet low in whole grains, at 193,559.8 (95% UI: 47,220.6 to 376,037.2). The low SDI region had the lowest number of DALYs observed, at 41,771 (95% UI: 9,658.2 to 80,100.4). However, the low SDI region had the highest ASMR [0.34 (95% UI: 0.09 to 0.64)] and ASDR [8.12 (95% UI: 1.962 to 15.45)] per 100,000 population, respectively. In contrast, the high-middle SDI region had the lowest ASMR [0.22 (95% UI: 0.05 to 0.41)] and ASDR [4.98 (95% UI: 1.23 to 9.78)] per 100,000 population, respectively. Between 1990 and 2021, the only region where ASMR decreased was the low SDI region, with an EAPC of −0.41 (95% CI: −0.51 to −0.31), while all other regions showed an increase. The high SDI region exhibited the most significant increase, with an EAPC of 1.5 (95% CI: 1.39 to 1.61). For ASDR, decreases were observed in the low SDI and high-middle SDI regions, with EAPCs of −0.5 (95% CI: −0.57 to −0.43) and −0.07 (95% CI: −0.19 to 0.05), respectively. All other regions showed an increase, with the high SDI region again demonstrating the most notable rise, with an EAPC of 1.2 (95% CI: 1.11 to 1.3) (Table 1; Figures 1, 2).

At the regional level, in 2021, East Asia had the highest number of CKD deaths [4,999.3 (95% UI: 1,282.9 to 9,456.7)] and DALYs [120,859.4 (95% UI: 29,793.1 to 239,415.2)] attributable to diet low in whole grains, followed by South Asia and high-income North America. In 2021, Eastern Europe had the lowest ASMR [0.06 (95% UI: 0.01 to 0.12)] and ASDR [2.3 (95% UI: 0.55 to 4.78)], while Andean Latin America had the highest ASMR [0.63 (95% UI: 0.16 to 1.16)] and Central Sub-Saharan Africa had the highest ASDR [14.84 (95% UI: 3.84 to 30.49)]. Central Europe showed the largest decreases in ASMR and ASDR, with an EAPC of −0.91 (95% CI: −1.11 to −0.71) for ASMR and −0.87 (95% CI: −0.98 to −0.77) for ASDR. In contrast, high-income North America exhibited the most significant increases, with an EAPC of 2.69 (95% CI: 2.49 to 2.9) for ASMR and 2.25 (95% CI: 2.07 to 2.43) for ASDR (Table 1; Figure 2).

### National level

**Figure 3:**
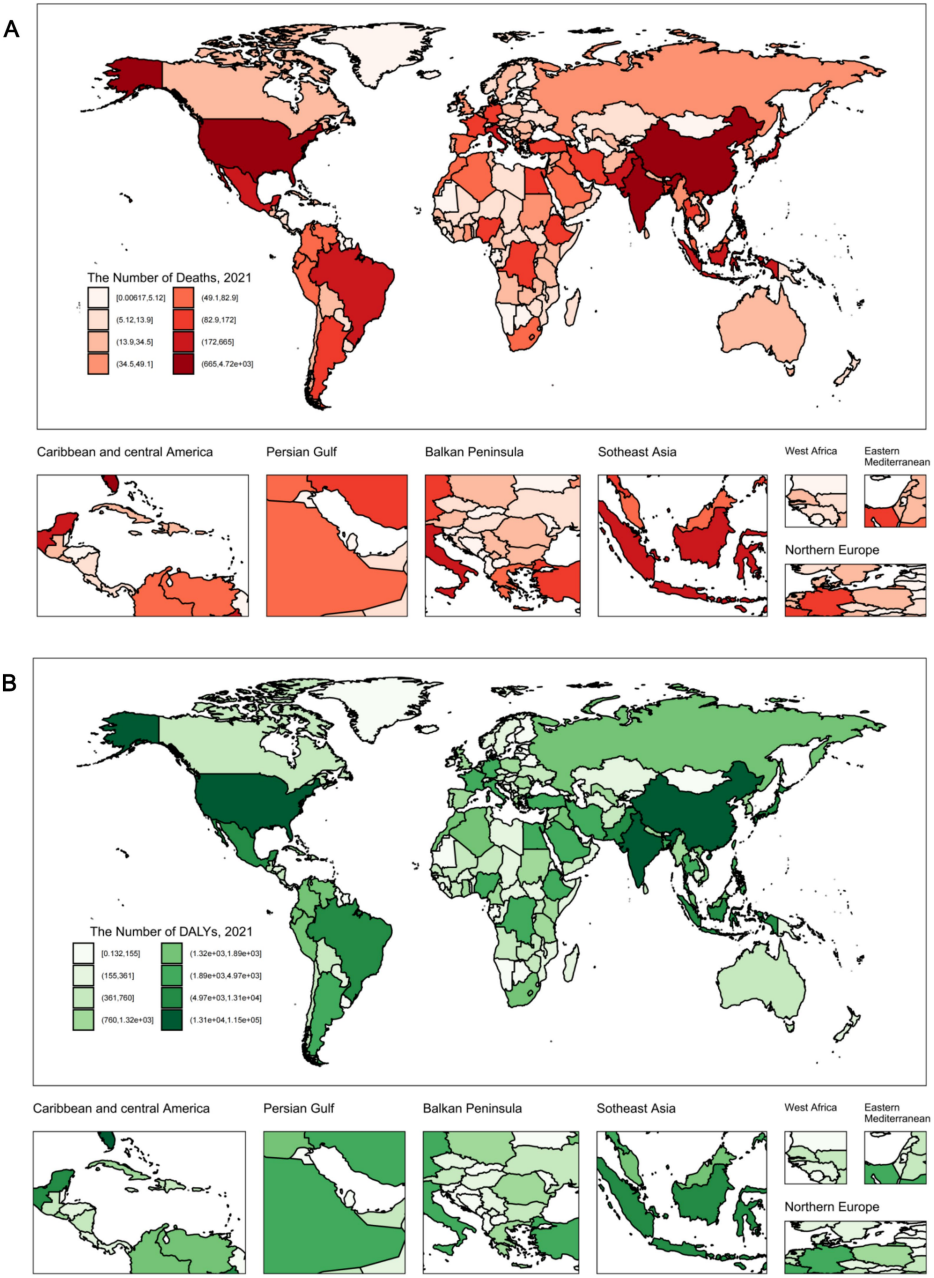
The CKD burden attributable to diet low in whole grains in 204 countries and territories. **(A)** The number of deaths in 2021. **(B)** The number of DALYs in 2021. DALYs, disability-adjusted life years.

**Figure 4:**
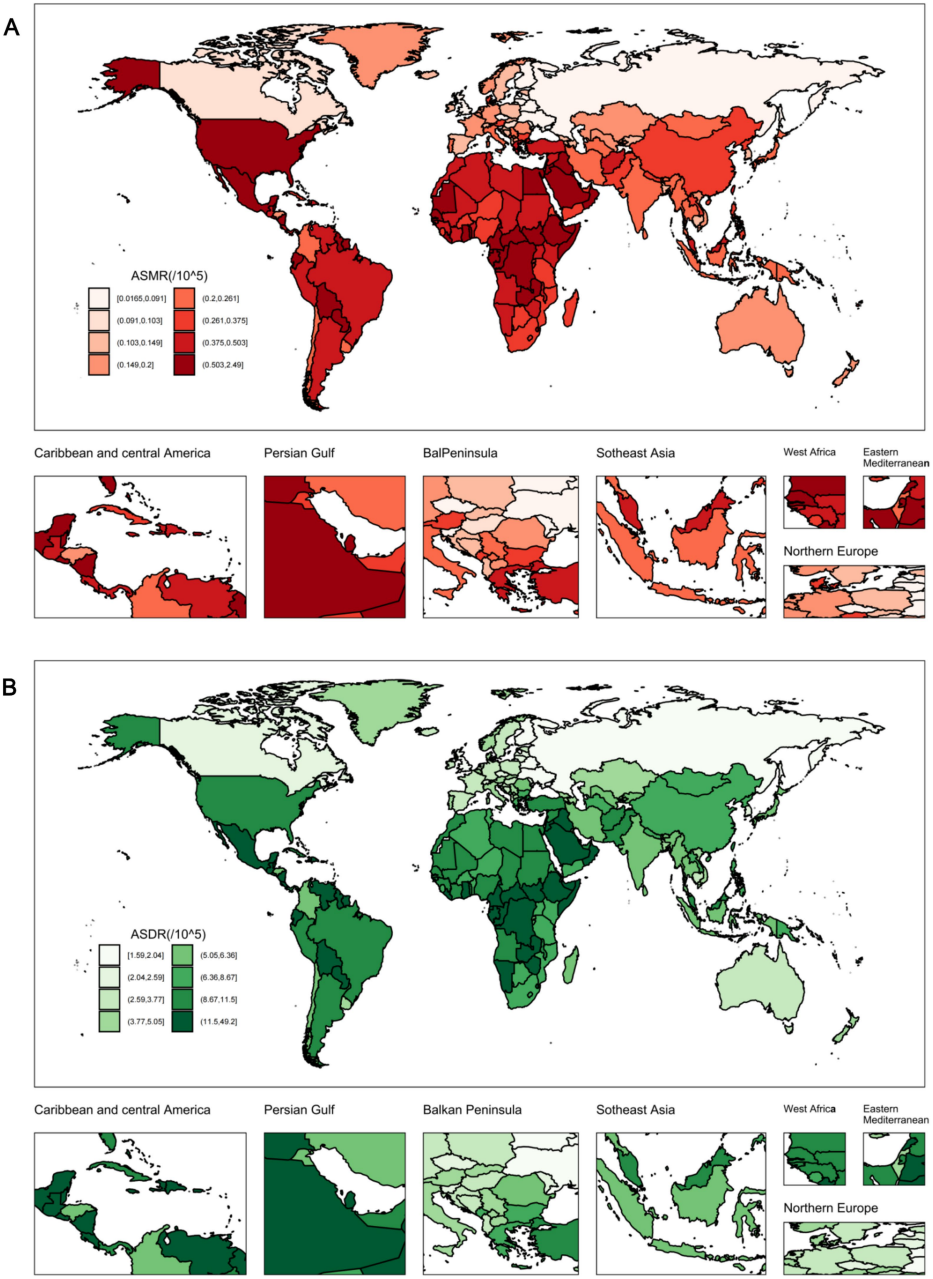
The CKD burden attributable to diet low in whole grains in 204 countries and territories. **(A)** The ASMR in 2021. **(B)** The ASDR in 2021. ASMR, Age-standardized mortality rate; ASDR, Age-standardized DALY rate.

At the national level, in 2021, China had the highest number of CKD deaths [4,721.8 (95% UI: 1,210.1 to 9,005.7)] and DALYs [114,616 (95% UI: 28,230.6 to 226,895.3)] attributable to diet low in whole grains. The number of deaths increased by 159% compared to 1990, and DALYs increased by 117% from 1990 levels. The United States of America had the next highest number of deaths, at 2,380.1 (95% UI: 622.2 to 4,371.8), while India had the second highest number of DALYs, at 68,501.9 (95% UI: 16,880.8 to 139,740.1). In contrast, Tokelau, Niue, Nauru, Greenland, and Tuvalu had the lowest levels (Figure 3; Supplementary Tables S1, S2). In 2021, American Samoa had the highest ASMR [2.2 (95% UI: 0.5 to 4)] per 100,000 population, followed by Niue, Fiji, the North Mariana Islands, Micronesia, the Marshall Islands, and Palau. Concurrently, American Samoa also had the highest ASDR, at 45.2 (95% UI: 11.4 to 85.1) in 2021, with Niue, Micronesia, the Marshall Islands, Fiji, Mauritius, Nauru, the North Mariana Islands, Palau, and Saudi Arabia following, each with an ASDR exceeding 25 per 100,000 population (Figure 4; Supplementary Tables S1, S2). Notably, between 1990 and 2021, Ukraine had the highest increase in ASMR, with an EAPC of 13.36 (95% CI: 11.43 to 15.31), while the United Arab Emirates had the highest increase in ASDR, with an EAPC of 3.77 (95% CI: 3.14 to 4.39). In contrast, from 1990 to 2021, the Republic of Poland had the fastest decline in ASMR, with an EAPC of −3.19 (95% CI: −3.82 to −2.55), followed by the Republic of Maldives, the Federal Democratic Republic of Ethiopia, the Republic of Cyprus, and the Solomon Islands. The Republic of Maldives had the fastest decline in ASDR, with an EAPC of −3.3 (95% CI: −3.52 to −3.07), followed by the Republic of Poland, the Federal Democratic Republic of Ethiopia, the Republic of Rwanda, and the Grand Duchy of Luxembourg (Supplementary Tables S1, S2).

### Age and sex patterns

**Figure 5:**
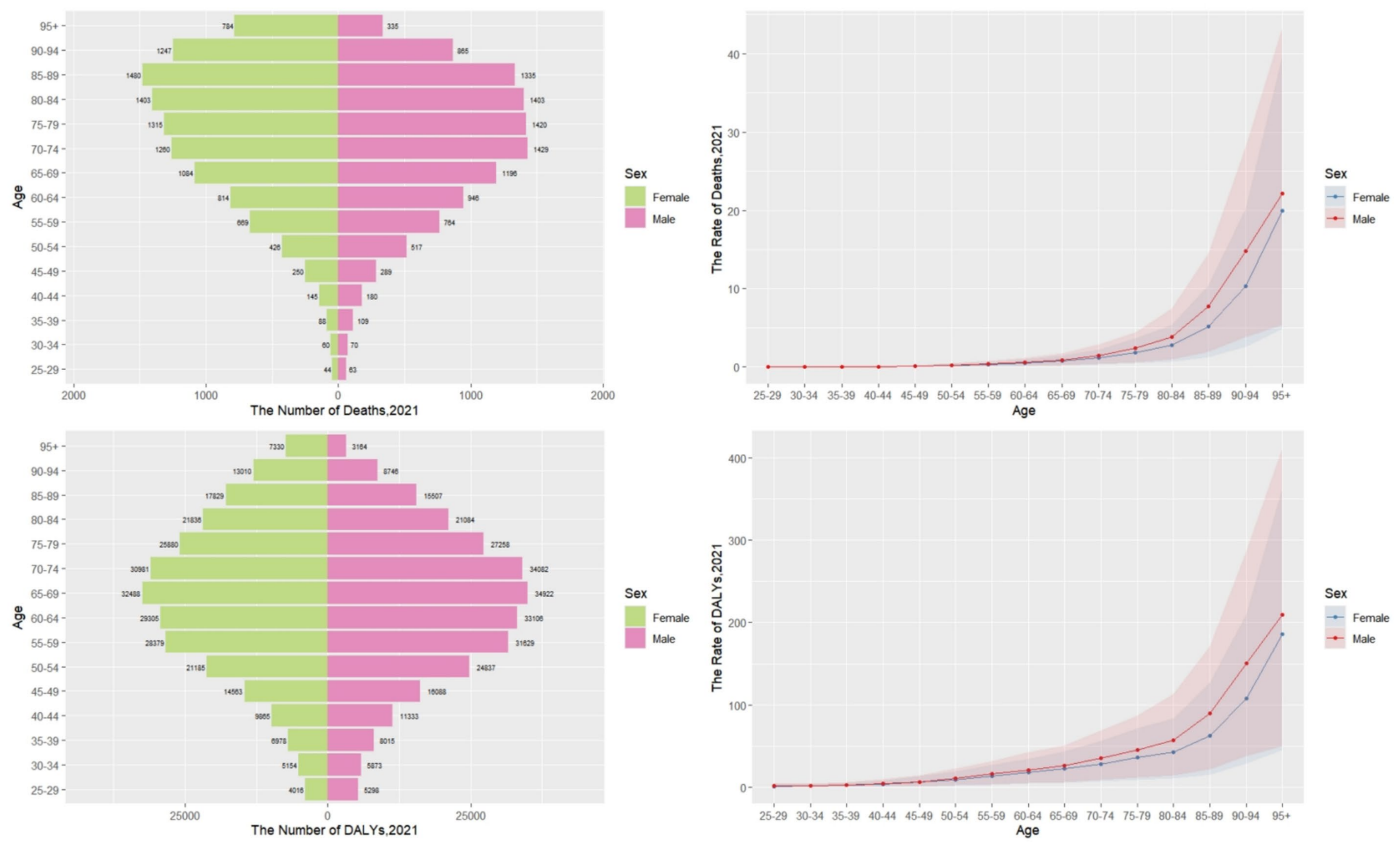
Global age- and sex-specific temporal trends in deaths and DALYs rates per 100,000 population for CKD attributable to diet low in whole grains, 2021.

In 2021, the global distribution of CKD deaths attributable to diet low in whole grains peaked within the 70–74 years age group. Mortality counts increased progressively with advancing age among individuals <75 years but decreased steadily in those ≥75 years. Similarly, CKD DALYs attributable to diet low in whole grains were highest in the 65–69 years cohort, exhibiting an age-dependent increase before 70 years and subsequent decline beyond this threshold (Figure 5).

**Figure 6:**
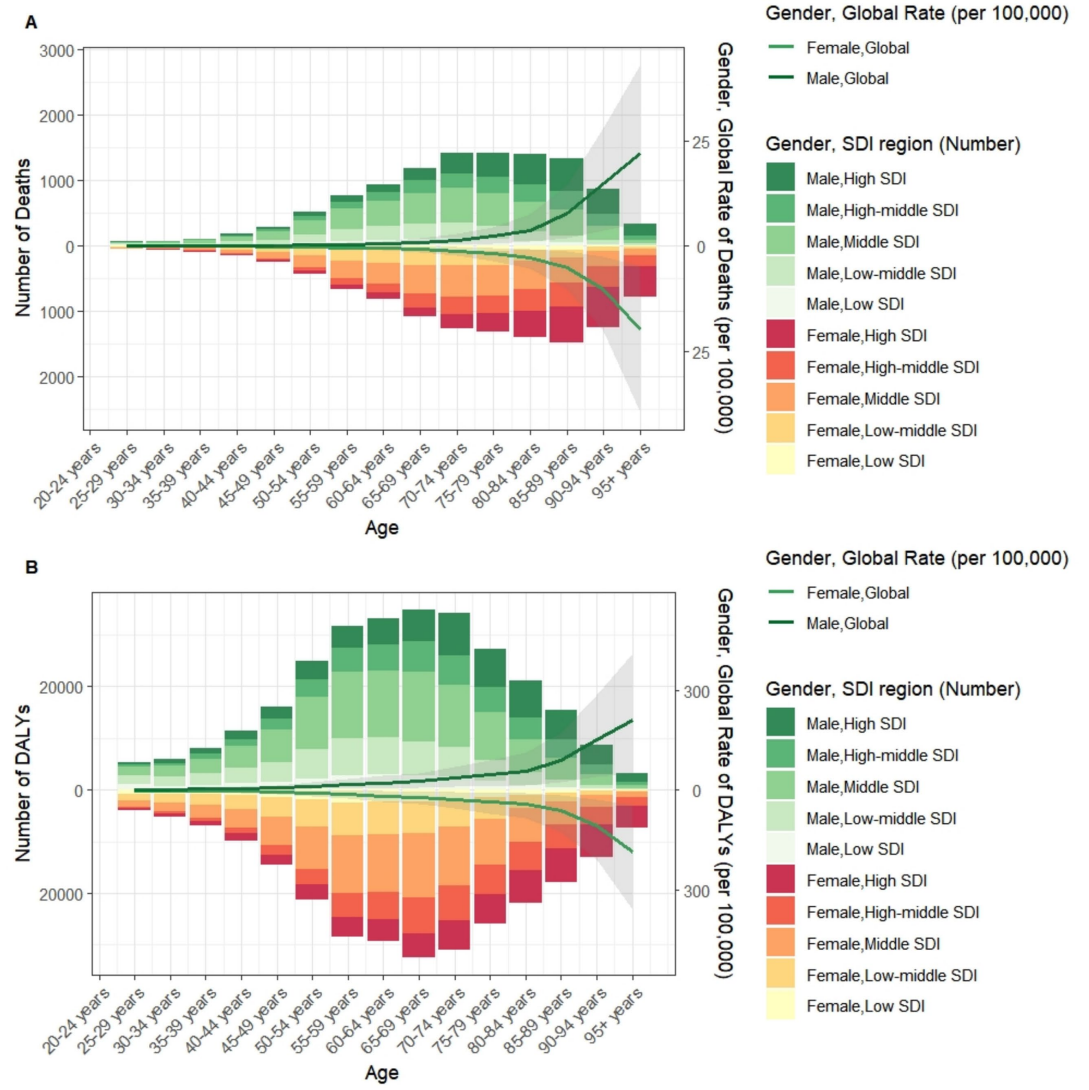
Number and rate of CKD deaths **(A)** and DALYs **(B)** attributable to diet low in whole grains by age group and SDI region in 2021. DALYs, Disability-adjusted life years.

**Figure 7:**
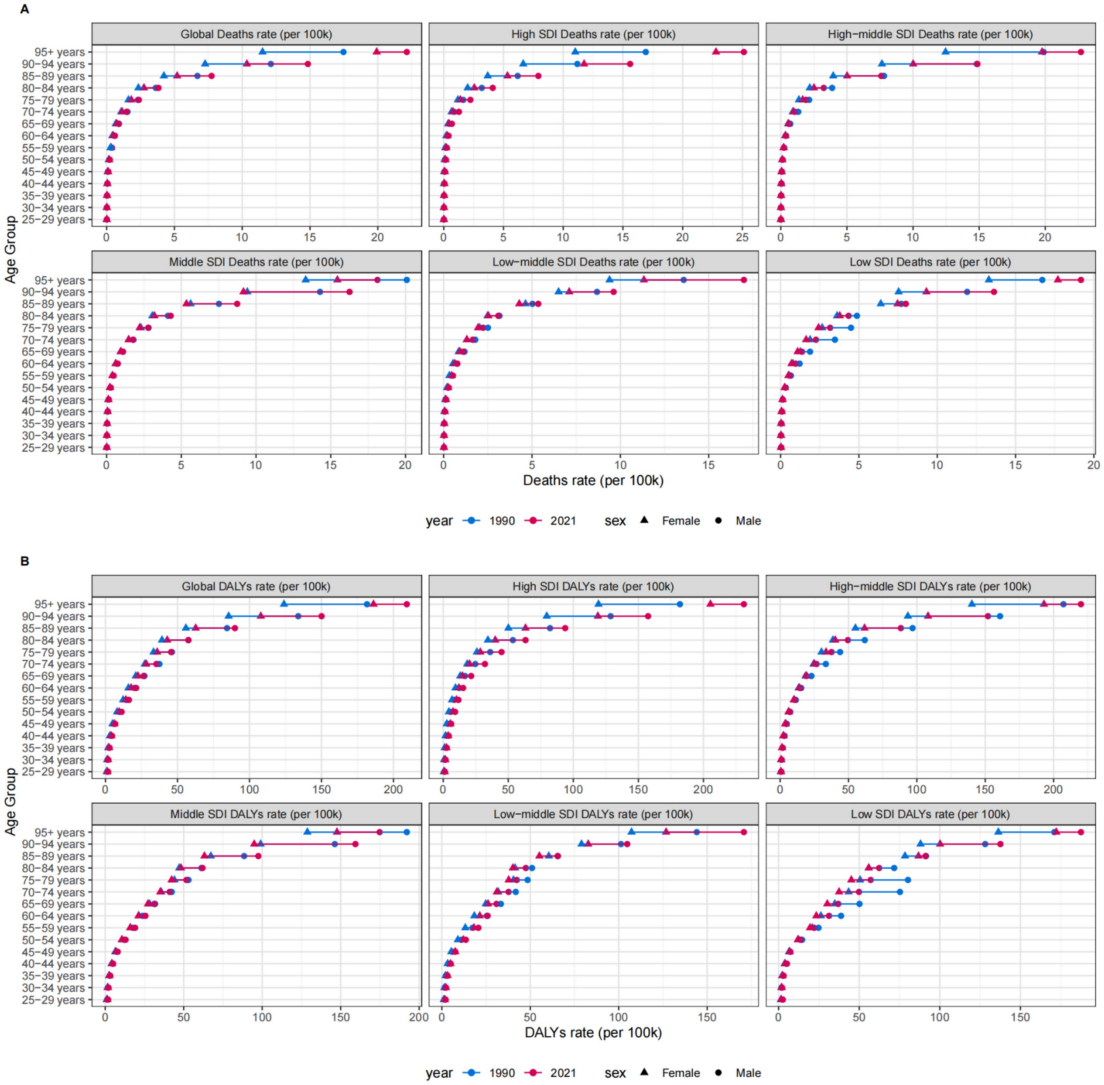
ASMR **(A)** and ASDR **(B)** of CKD attributable to diet low in whole grains by sex, age group, and social development index, 1990 and 2021. ASMR, Age-standardized mortality rate; ASDR, Age-standardized DALY rate.

For individuals under 60 years of age, the highest numbers of deaths and DALYs were observed in middle SDI regions. For those aged between 60 and 85 years, the highest figures were recorded in middle SDI regions. Finally, for individuals aged 85 years and older, the highest numbers were seen in high SDI regions. Overall, global age-specific mortality rates and DALY rates increased with age (Figure 6). Sex-based analyses revealed no significant differences between males and females before age 70–74 years. However, both ASMR and ASDR became substantially elevated in males from age 75 years onward, with this gender disparity widening progressively and peaking in the ≥95 years cohort (Figure 7).

### The association between the SDI and the CKD burden attributable to diet low in whole grains

**Figure 8:**
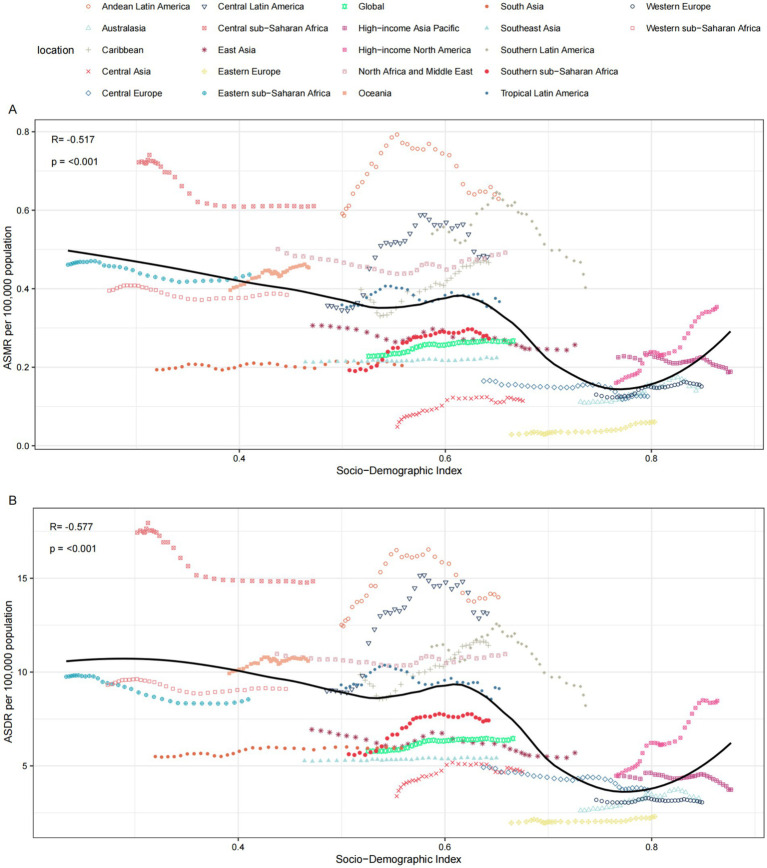
The relationships between the SDI and the CKD burdens attributable to diet low in whole grains among the 21 GBD regions between 1990 and 2021. **(A)** The association between CKD attributable to diet low in whole grains ASMR and SDI among 21 GBD regions. **(B)** The association between CKD attributable to diet low in whole grains ASDR and SDI among 21 GBD regions. ASMR, Age-standardized mortality rate; ASDR, Age-standardized DALY rate.

Overall, there exists a non-linear “W”-shaped correlation between the SDI and the ASMR and ASDR of CKD attributable to diet low in whole grains. As the SDI increases, both ASMR and ASDR first show a gradual downward trend, followed by a brief slow rise, then a rapid decline to a minimum of approximately 0.73, and finally an upward trend again. Based on the SDI from 1990 to 2021, regions such as Central sub-Saharan Africa, Andean Latin America, Central Latin America, North Africa and the Middle East, and Southern Latin America exhibited higher ASMR and ASDR than expected. In contrast, from 1990 to 2021, the burden in Western sub-Saharan Africa, Eastern sub-Saharan Africa, South Asia, Southern sub-Saharan Africa, Eastern Europe, Central Asia, Southeast Asia, and Western Europe was lower than expected (Figure 8).

### Cross-country health inequality analysis

**Figure 9:**
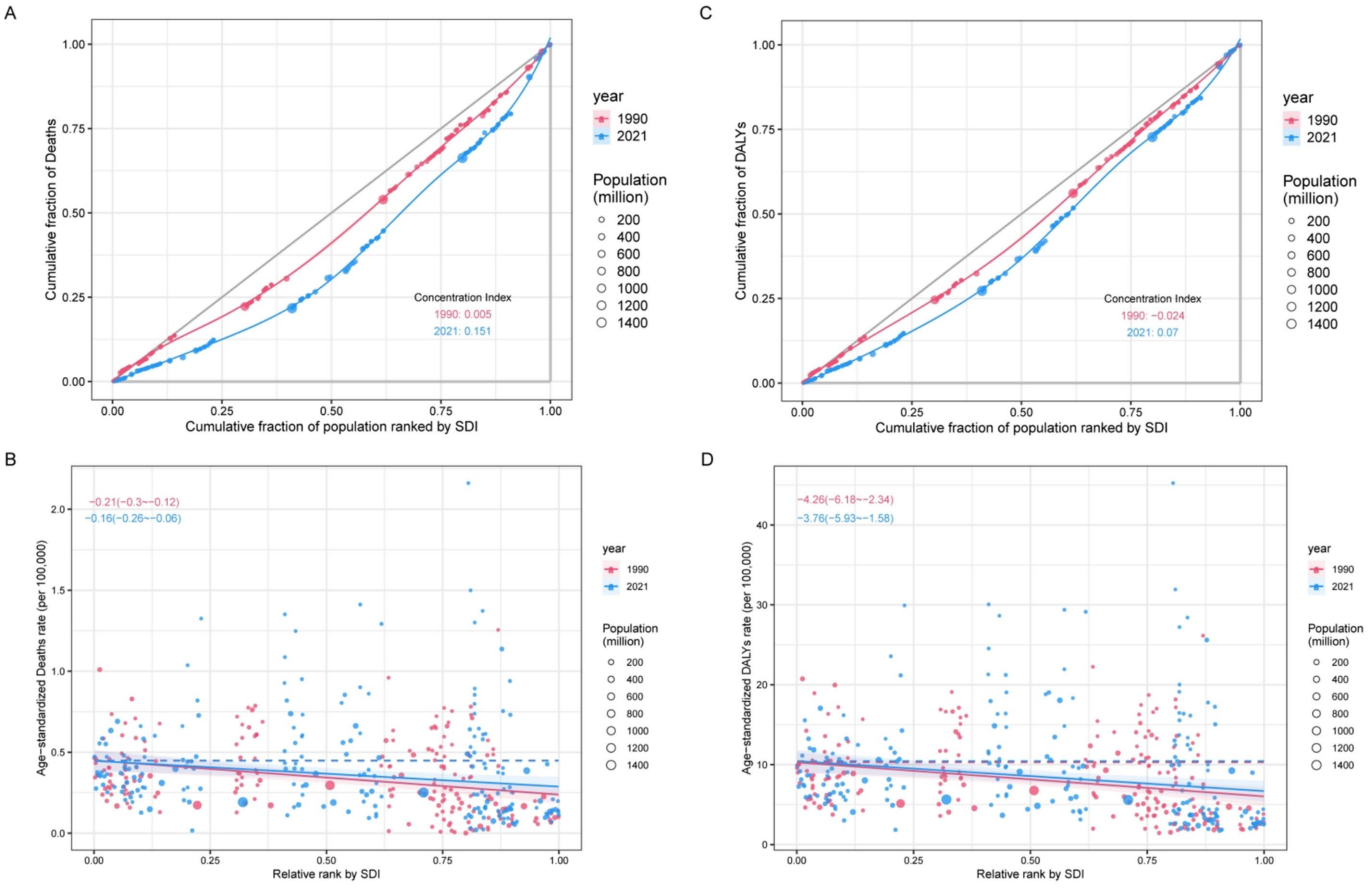
Health inequality regression curves and concentration curves for the deaths **(A,B)** and DALYs **(C,D)** of CKD attributable to diet low in whole grains. DALYs, disability-adjusted life years.

The findings indicate that there exists relative inequality in the mortality and DALYs burdens of CKD attributable to diet low in whole grains. The burden of CKD due to diet low in whole grains is concentrated in impoverished regions (Figure 9). A comparison of data from 1990 and 2021 reveals a reduction in health inequalities. The concentration index for mortality changed from 0.005 in 1990 to 0.151 in 2021 (Figure 9A), and the SII was −0.21 (95% CI: −0.3 to −0.12) in 1990 and −0.16 (95% CI: −0.26 to −0.06) in 2021 (Figure 9B). The concentration index for DALYs ranged from −0.024 in 1990 to 0.07 in 2021 (Figure 9C). The SII for DALYs decreased from −4.26 (95% CI: −6.18 to −2.34) in 1990 to −3.76 (95% CI: −5.93 to −1.58) in 2021 (Figure 9D). The observed decrease in the negative values of the two slope indices indicates a narrowing of the absolute burden gap between low and high SDI regions over time.

### ARIMA predictive analysis on CKD burden attributed to diet low in whole grains to 2050

**Figure 10:**
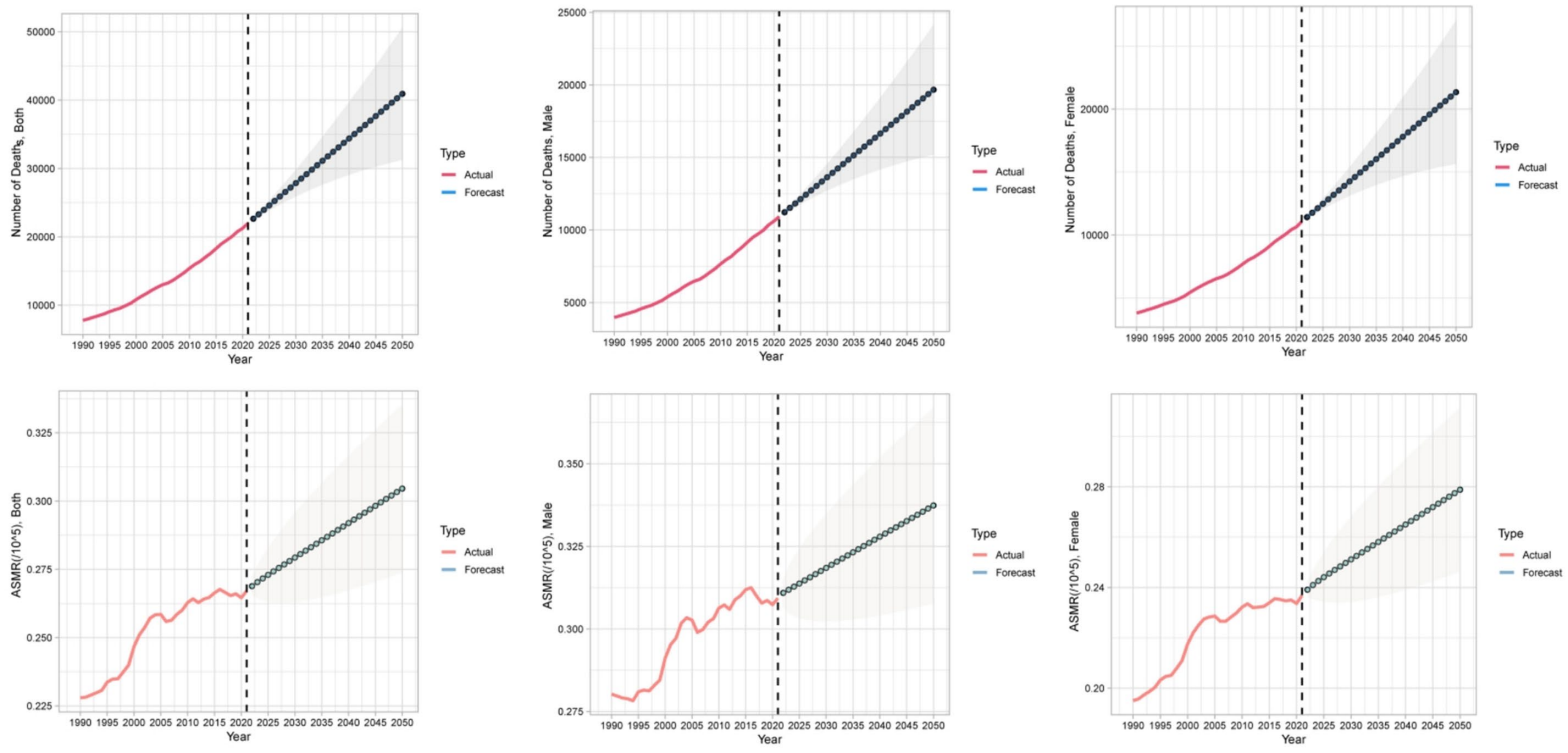
Temporal trends in global death numbers and ASMR of CKD attributable to diet low in whole grains, 1990–2050. ASMR, Age-standardized mortality rate.

**Figure 11:**
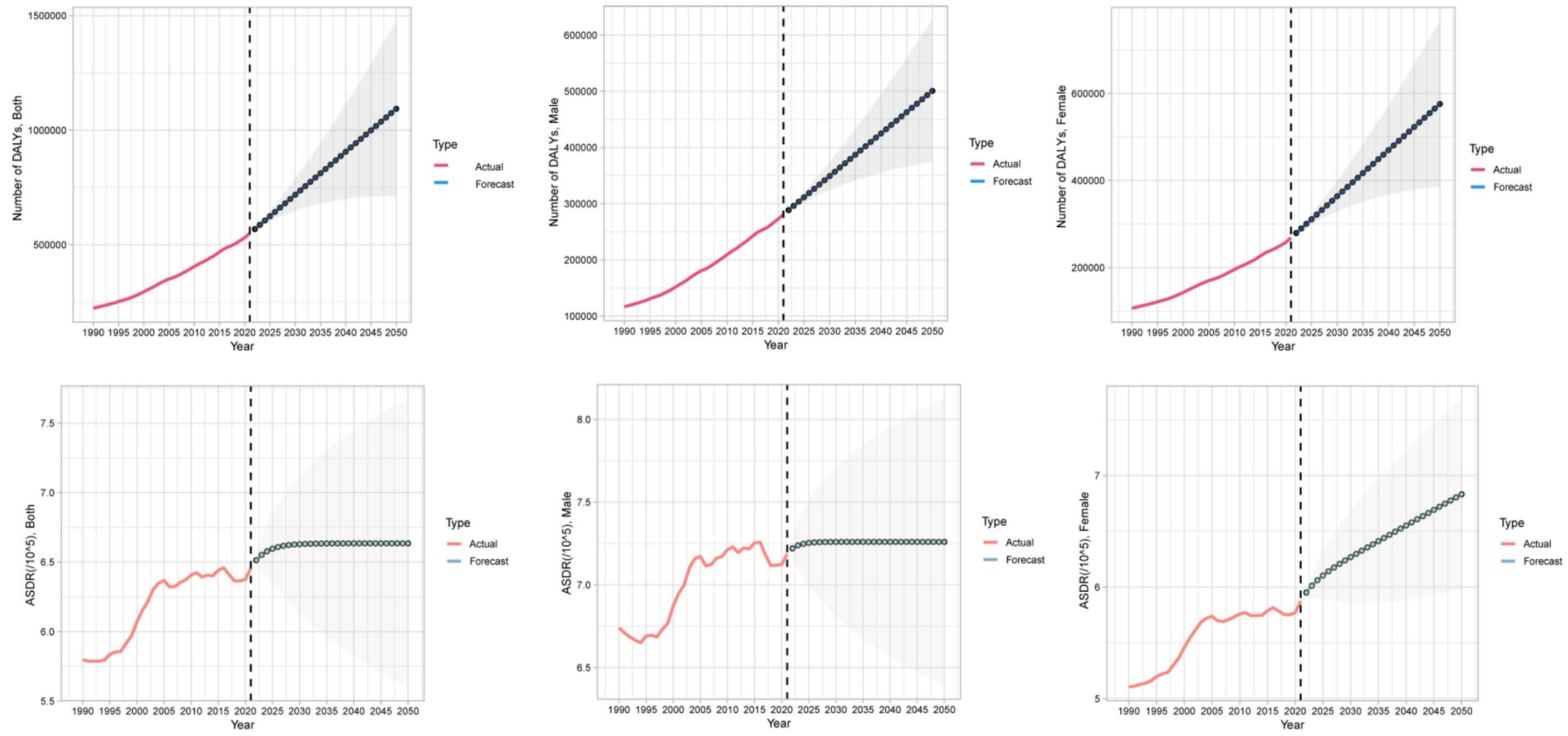
Temporal trends in global DALYs numbers and ASDR of CKD attributable to diet low in whole grains, 1990–2050. DALYs, Disability-adjusted life years; ASDR, Age-standardized DALY rate.

We applied the auto.arima() function to global data on CKD attributable to diet low in whole grains from 1990 to 2021, and predicted trends over the next 30 years, selecting the optimal models for the number of deaths, DALYs, ASMR, and ASDR. The Ljung-Box test confirmed that the model residuals were white noise (Supplementary Table S3). According to the ARIMA model results, over the next 30 years, the global number of deaths from CKD due to diet low in whole grains and the ASMR will continue to rise. The global number of DALYs from CKD due to diet low in whole grains will also keep increasing, and the ASDR in females will show an upward trend, while the ASDR in the total population and males will remain relatively stable (Figures 10, 11).

## Discussion

This comprehensive analysis reveals a substantial and growing global burden of chronic kidney disease attributable to diet low in whole grains, with deaths increasing by 183% and DALYs by 146% between 1990 and 2021.

The persistent upward trajectory in age-standardized rates (ASMR EAPC 0.58%; ASDR EAPC 0.41%) underscores a critical public health challenge that has not been mitigated by contemporary renal care paradigms (18–20). Our data demonstrate complex socioeconomic patterning of this burden, characterized by three principal phenomena: first, East Asia and the concentration of absolute burden in middle SDI regions reflects population demographics and dietary transition stages; second, the disproportionate ASMR and ASDR burden in low SDI regions (ASDR 8.12 vs. 4.98/100,000 in high-middle SDI) highlights healthcare access disparities; third, the unexpected W-shaped SDI association reveals nonlinear development-burden relationships (21). This tripartite inequality pattern—where Andean Latin America and Central Sub-Saharan Africa exhibited rates 2–3 times higher than Eastern Europe—emphasizes how local food environments interact with socioeconomic determinants to shape renal outcomes (22, 23).

Further analysis revealed that East Asia bears the heaviest burden of kidney disease associated with whole grain intake. The primary reasons include a rapid dietary transition, characterized by the widespread introduction of high-protein diets typical of Western countries, which has shifted local eating habits. This is coupled with a high degree of staple food refinement leading to the loss of beneficial components in whole grains, and a lack of emphasis on increasing whole grain consumption to match these dietary changes. Consequently, whole grain intake in the region remains substantially below recommended levels (24). Concurrently, uneven coverage of early screening for chronic diseases in health policies acts as an additional driver exacerbating the disease burden (25). In contrast, low to middle SDI regions face multifaceted challenges: weak whole grain production and supply systems, insufficient dietary diversity, and limited primary healthcare capacity. This results in a dual deficiency in both nutritional intervention and kidney disease management, thereby amplifying the health burden. These disparities suggest that East Asia should focus on dietary pattern restructuring and policy guidance, whereas low to middle SDI regions require strengthened support for whole grain supply chains and the integration of nutritional interventions into primary healthcare systems.

The demographic stratification provides further pathophysiological insights. The peak burden at ages 65–74 years aligns with the cumulative impact of prolonged nutritional inadequacy on renal function (26), while the emergent male predominance beyond age 75 (gender gap exceeding 20% in ≥95y cohort) likely reflects biological susceptibility compounded by lifestyle factors (27). Our projection models amplify concern: ARIMA forecasts indicate an 86% mortality increase by 2050, with females experiencing a unique 16% ASDR rise despite stable male rates—a divergence potentially tied to postmenopausal metabolic alterations affecting nutrient processing (28). These trajectories highlight an impending demographic crisis where aging populations in High SDI regions and nutritionally vulnerable communities in Low SDI areas will face compounding renal risks.

Confronted with these stratified risks, a multi-tiered public health response is essential. For high-SDI regions with aging populations, policy should focus on integrating nutritional screening and personalized dietary guidance into routine geriatric and primary care, specifically promoting whole grain consumption to mitigate CKD progression. This requires strengthening early screening systems for CKD and its risk factors like hypertension and diabetes within primary care settings. For low- and middle-SDI regions, the priority is building foundational capacity, which includes fortifying local whole grain supply chains to improve accessibility and affordability, and task-sharing basic nutrition interventions and CKD management to community health workers to overcome workforce shortages. Technologically, implementing CKD dashboards that use risk stratification tools like the Kidney Failure Risk Equation (KFRE) can enable healthcare systems to identify high-risk individuals for targeted intervention, while telehealth and digital health tools can extend care reach. Ultimately, effectively addressing the growing burden requires moving away from a one-size-fits-all approach and instead implementing region-specific strategies that combine food system interventions with the integration of CKD prevention and management into primary healthcare, guided by a framework like the Chronic Care Model to ensure proactive, patient-centered support.

The mechanisms underlying whole grains’ renal protection warrant emphasis. The documented benefits—dietary fiber’s uremic toxin modulation (29, 30), magnesium’s blood pressure regulation (31), and phenolic compounds’ oxidative stress reduction (32)—provide biological plausibility for our population-level findings. This mechanistic understanding strengthens the causal inference between suboptimal intake and CKD progression, particularly given the dose-dependent relationships observed in cohort studies (33). ​​The dietary fiber in grains contributes to renal health through multiple pathways. The underlying mechanisms are elaborated as follows: it promotes fecal excretion, thereby increasing nitrogen elimination and reducing serum nitrogen levels (34). Furthermore, fiber intake alters gut microbiota composition, leading to decreased levels of pro-inflammatory cytokines and uremic toxins (3, 35, 36). The fermentation of dietary fiber produces short-chain fatty acids (SCFAs), which can mitigate systemic inflammation, maintain mucosal barrier integrity, and modulate immune and anti-inflammatory responses via T-cell regulation (37). Supporting this, a 6.1-year prospective cohort study by Mirmiran et al. involving 1,630 participants found an inverse association between total fiber intake and CKD incidence, with each daily 5-gram increment in dietary fiber from food associated with an 11% lower risk of CKD (38). Additionally, magnesium—another key component of whole grains—confers renal protection. It helps protect the kidneys from phosphate-induced injury by inhibiting phosphate-mediated renal tubular cell apoptosis and reducing calcium phosphate crystal formation on the tubular epithelium (39). Experimental evidence reinforces this: in a CKD mouse model, a low-magnesium, high-phosphorus diet administered for 6 weeks downregulated renal *α*-klotho expression and significantly exacerbated the tubular damage and interstitial fibrosis induced by high phosphorus intake (40). Similarly, rats fed a magnesium-deficient diet exhibited renal tubulointerstitial injury, whereas magnesium supplementation was shown to improve their renal function (41).

Moreover, the strong confounding effects of unhealthy dietary patterns, such as those high in salt and sugar, must be carefully considered. In practice, low whole-grain intake often coexists with high consumption of processed foods and elevated sodium and sugar intake—the latter two being established independent risk factors for hypertension, diabetes, and kidney injury. This collinearity may exaggerate or confound the causal relationship between whole grains per se and renal outcomes. The present study utilized the GBD database and its analytical framework incorporated statistical adjustments for key confounders—including age, sex, blood pressure, and fasting blood glucose—through multivariate regression models and Bayesian tools such as DisMod-MR 2.1.

On the other hand, while the absolute health inequality, as measured by the SII, showed a slight improvement during the period 1990–2021, the relative inequality in mortality burden, reflected by the CI, became more pronounced. This seemingly paradoxical phenomenon can be reconciled by considering the observed “W-shaped” SDI-burden relationship. This “W-shaped” curve describes a non-linear association between the overall burden level and societal development, capturing the differences in the combination of disease risks at various stages of SDI. In contrast, the CI and SII measure the distribution of the burden along the socioeconomic gradient at specific points in time. The increasingly negative CI indicates that low-SDI regions bear a progressively larger proportion of the total burden relative to their population share. Concurrently, the improvement in the SII suggests that the absolute rate difference between the most impoverished and the most affluent regions is narrowing, a change potentially attributable to broader public health advancements in low-SDI areas.

Methodological considerations contextualize these findings. The GBD framework provides standardized global estimates but carries inherent uncertainty from heterogeneous input data, particularly in regions with limited vital registration (42). Our ARIMA modeling captured linear trends effectively but cannot account for unforeseen nutritional transitions or policy disruptions. To address the limitations outlined above, future research should focus on integrating macro-level data with local surveillance data to enhance data quality, and explore the application of nonlinear modeling approaches, such as machine learning, or causal inference methods to better capture complex influences. Ultimately, combining these advances with large-scale prospective cohort studies to validate associations and elucidate underlying mechanisms at the individual level will provide critical evidence for developing personalized dietary interventions.

Given that long-term forecasts may be influenced by unforeseen structural changes (such as dramatic shifts in global dietary policies or major breakthroughs in food technology), we implemented the following procedures to enhance the robustness of our results. First, the rationale is based on historical trends; our projections are strictly grounded in the observed, relatively stable trajectory of disease burden change over the past 32 years (1990–2021). Model diagnostics confirmed the absence of significant structural breaks in the historical data that would invalidate the model. Second, we conducted sensitivity analyses to assess the influence of model choice and the time window used. Specifically, we performed supplementary analyses including: (a) modeling with an alternative time window by refitting the ARIMA model using the most recent 15 years of data (2007–2021) and projecting to 2050 to examine if recent trends alter the long-term projection direction; and (b) comparison with a simple extrapolation method by contrasting the ARIMA model projections with those from the Holt-Winters exponential smoothing method, a non-parametric approach that captures trend and seasonality. The relative difference between the point estimates for 2050 from the two methods was less than 10%, and the direction of the trends was completely consistent. This demonstrates that our core predictive conclusions (e.g., the upward or downward trend in disease burden) are robust under different modeling assumptions. Based on this analysis, we conclude that, assuming no fundamental reversal in current risk factor exposure trends and disease patterns, projections to 2050 can provide a valuable reference for medium- to long-term public health planning.

Over the 1990–2021 period, the global burden of CKD attributable to diet low in whole grains demonstrated a persistent upward trajectory, with pronounced impacts observed in middle SDI regions, males, and older adults. Notably, low SDI regions bore disproportionately high ASMR and ASDR, whereas high SDI regions exhibited the most rapid increase rates. These findings underscore an urgent need to implement targeted CKD prevention strategies and health education initiatives for high-risk populations to enhance public understanding and intake of whole grains. Concurrently, intensified screening and interventions in these demographics are imperative to mitigate rising CKD mortality risks associated with insufficient whole grain consumption. We recommend three synergistic approaches: agricultural policies promoting whole-grain accessibility in food-insecure regions; clinical protocols incorporating dietary metrics into renal risk assessment; and culturally adapted consumer education targeting high-risk populations. Implementing such multifaceted strategies offers substantial potential to mitigate this growing threat to global renal health.

## Data Availability

The raw data supporting the conclusions of this article will be made available by the authors, without undue reservation.

## Footnotes

1
https://vizhub.healthdata.org/gbd-results/
