# Supplementary material for: The burden of chronic kidney disease attributable to diet low in whole grains from 1990 to 2021: a global, regional and national analysis
Source: Front Nutr. 2026 Feb 20;13:1684886. doi: 10.3389/fnut.2026.1684886 (PMC12962927; doi:10.3389/fnut.2026.1684886)
Supplement: Supplementary file 1 [file Data_Sheet_1.PDF]

Table S1 The deaths burden of CKD attributable to diet low in whole grains in 204 countries and territories, 1990 - 2021.

Table S2 The DALYs burden of CKD attributable to diet low in whole grains in 204 countries and territories, 1990 - 2021.

Table S3 The (p, d, q), AIC, and Ljung-Box (P-value) values of the ARIMA model for the CKD burdens attributable to diet low in whole grains in globally.

Figure S1 Data Retrieval and Download Steps

TableS1 The deaths burden of CKD attributable to diet low in whole grains in 204 countries and territories, 1990 - 2021.

location	1990		2021		EAPC_95%CI
	Number(95% UI)	ASR(95% UI)	Number(95% UI)	ASR(95% UI)	
American Samoa	0.1 (0-0.2)	0.8 (0.2-1.4)	0.9 (0.2-1.6)	2.2 (0.5-4)	3.65 (3.39 to 3.92)
Antigua and Barbuda	0.3 (0.1-0.5)	0.5 (0.1-1)	0.7 (0.2-1.3)	0.7 (0.2-1.3)	1.66 (1.36 to 1.95)
Arab Republic of Egypt	132.9 (31.4-269.3)	0.8 (0.2-1.6)	335 (89.8-672.8)	0.7 (0.2-1.5)	-0.1 (-0.21 to 0.01)
Argentine Republic	197.9 (50.2-377.5)	0.7 (0.2-1.3)	284.2 (74-539.3)	0.5 (0.1-0.9)	-0.63 (-1.01 to -0.25)
Australia	20.3 (4.6-40.3)	0.1 (0-0.2)	79.6 (18.8-153.4)	0.1 (0-0.3)	1.53 (1.25 to 1.82)
Barbados	1.4 (0.4-2.5)	0.5 (0.1-0.9)	3 (0.8-5.5)	0.6 (0.2-1.1)	1.38 (1.07 to 1.7)
Belize	0.4 (0.1-0.8)	0.5 (0.1-0.8)	1.9 (0.5-3.3)	0.7 (0.2-1.1)	1.4 (0.95 to 1.86)
Bermuda	0.3 (0.1-0.5)	0.5 (0.1-0.9)	0.6 (0.2-1.1)	0.4 (0.1-0.7)	-0.06 (-0.36 to 0.25)
Bolivarian Republic of Venezuela	18.9 (4.8-37.9)	0.2 (0.1-0.4)	116 (27.4-233.6)	0.4 (0.1-0.8)	1.72 (1.26 to 2.19)
Bosnia and Herzegovina	3.9 (0.9-8.7)	0.1 (0-0.2)	6.1 (1.5-12.8)	0.1 (0-0.2)	-0.61 (-0.98 to -0.24)
Brunei Darussalam	0.3 (0.1-0.6)	0.4 (0.1-0.8)	0.8 (0.2-1.6)	0.4 (0.1-0.7)	0.4 (0.13 to 0.67)
Burkina Faso	11.4 (2.8-23.1)	0.4 (0.1-0.7)	24.6 (5.6-49.3)	0.4 (0.1-0.7)	-0.05 (-0.11 to 0.02)
Canada	29 (7.6-56.8)	0.1 (0-0.2)	80.4 (19.2-154.5)	0.1 (0-0.2)	0.49 (0.2 to 0.79)
Central African Republic	6.9 (1.6-14.1)	0.7 (0.2-1.5)	11 (2.5-23.8)	0.6 (0.2-1.2)	-0.77 (-0.83 to -0.71)
Commonwealth of Dominica	0.3 (0.1-0.6)	0.6 (0.2-1)	0.6 (0.2-1.2)	0.8 (0.2-1.5)	1.23 (1.14 to 1.32)
Commonwealth of the Bahamas	0.8 (0.2-1.4)	0.5 (0.1-0.9)	2.4 (0.6-4.2)	0.6 (0.2-1.1)	1.2 (0.95 to 1.46)
Cook Islands	0 (0-0.1)	0.4 (0.1-0.8)	0.2 (0-0.3)	0.6 (0.2-1.1)	1.31 (1.2 to 1.42)
Czech Republic	12.8 (3.1-25.8)	0.1 (0-0.2)	14.7 (3.7-29.7)	0.1 (0-0.1)	-1.28 (-1.5 to -1.07)
Democratic People's Republic of Korea	43.3 (10-82.6)	0.3 (0.1-0.7)	83.2 (19.6-161.9)	0.3 (0.1-0.5)	-0.57 (-0.72 to -0.42)
Democratic Republic of Sao Tome and Principe	0.3 (0.1-0.6)	0.6 (0.1-1.2)	0.6 (0.1-1.2)	0.7 (0.2-1.4)	0.57 (0.5 to 0.64)
Democratic Republic of Timor-Leste	0.5 (0.1-0.9)	0.2 (0.1-0.5)	1.4 (0.3-2.9)	0.2 (0-0.4)	-0.63 (-0.97 to -0.29)
Democratic Republic of the Congo	98.3 (25.4-193.7)	0.8 (0.2-1.6)	205.7 (54.9-426.4)	0.7 (0.2-1.4)	-0.83 (-0.95 to -0.7)
Democratic Socialist Republic of Sri Lanka	18.5 (4.4-34.5)	0.2 (0.1-0.4)	41.5 (9.9-88)	0.2 (0-0.4)	-0.77 (-1 to -0.54)
Dominican Republic	11.4 (2.7-22.3)	0.4 (0.1-0.7)	39.1 (10.4-74.1)	0.4 (0.1-0.8)	1.13 (0.81 to 1.46)
Eastern Republic of Uruguay	8 (1.9-14.9)	0.2 (0-0.4)	15.4 (3.9-31.2)	0.2 (0.1-0.5)	0.66 (0.37 to 0.96)
Federal Democratic Republic of Ethiopia	152.2 (36.9-291.3)	1 (0.3-1.9)	215 (51.3-421.1)	0.6 (0.2-1.2)	-1.96 (-2.11 to -1.8)
Federal Democratic Republic of Nepal	15.2 (3.5-30)	0.2 (0-0.4)	46 (10.7-89.6)	0.2 (0.1-0.4)	0.49 (0.27 to 0.71)
Federal Republic of Germany	129.3 (33.9-264.8)	0.1 (0-0.2)	379.7 (90.5-770.3)	0.1 (0-0.3)	2.39 (1.87 to 2.92)
Federal Republic of Nigeria	124.6 (30.2-239.3)	0.4 (0.1-0.7)	220.7 (55.1-428.3)	0.3 (0.1-0.6)	-0.78 (-0.96 to -0.59)
Federal Republic of Somalia	7.6 (1.6-16.2)	0.5 (0.1-1)	20.3 (5.1-43.1)	0.5 (0.1-1)	0.16 (0.01 to 0.31)
Federated States of Micronesia	0.4 (0.1-0.8)	1 (0.3-2)	0.8 (0.2-1.6)	1.4 (0.3-2.6)	1.32 (1.13 to 1.52)
Federative Republic of Brazil	273.3 (66.7-495.5)	0.4 (0.1-0.7)	894.8 (229.7-1658.9)	0.4 (0.1-0.7)	0.02 (-0.15 to 0.19)
French Republic	132.9 (32.8-267.1)	0.1 (0-0.3)	303.8 (76.3-596.1)	0.1 (0-0.3)	0.31 (0.02 to 0.6)
Gabonese Republic	3.3 (0.7-6.8)	0.7 (0.2-1.4)	7.9 (1.9-16.2)	1 (0.2-1.9)	0.91 (0.68 to 1.14)
Georgia	1.6 (0.4-3.4)	0 (0-0.1)	6.2 (1.3-12.3)	0.1 (0-0.2)	4.57 (3.87 to 5.28)
Grand Duchy of Luxembourg	1 (0.2-2)	0.2 (0-0.4)	1.5 (0.4-3)	0.1 (0-0.2)	-1.46 (-1.82 to -1.09)
Greenland	0 (0-0.1)	0.1 (0-0.3)	0.1 (0-0.1)	0.1 (0-0.3)	0.05 (-0.09 to 0.19)
Grenada	0.5 (0.1-0.8)	0.6 (0.1-1.1)	1 (0.2-1.7)	0.9 (0.2-1.6)	1.77 (1.53 to 2)
Guam	0.3 (0.1-0.6)	0.6 (0.2-1.1)	0.9 (0.2-1.7)	0.4 (0.1-0.8)	-0.13 (-0.52 to 0.26)
Hashemite Kingdom of Jordan	5.4 (1.3-10.2)	0.5 (0.1-1)	36.1 (9.1-69)	0.6 (0.2-1.2)	1.01 (0.75 to 1.28)
Hellenic Republic	66.5 (17.5-135.7)	0.5 (0.1-1)	149.4 (37.5-294.1)	0.4 (0.1-0.9)	-0.99 (-1.97 to 0)
Hungary	9.4 (2.2-18.5)	0.1 (0-0.1)	20.6 (5-40.3)	0.1 (0-0.2)	2.3 (1.87 to 2.74)

Independent State of Papua New Guinea	3.4 (0.9-6.7)	0.2 (0.1-0.4)	8.7 (2-17.2)	0.2 (0-0.4)	-0.33 (-0.42 to -0.24)
Independent State of Samoa	0.5 (0.1-0.9)	0.6 (0.1-1.2)	1.4 (0.4-2.7)	1.1 (0.3-2.1)	2 (1.9 to 2.11)
Ireland	4.8 (1.1-9.4)	0.1 (0-0.3)	10.6 (2.6-20.7)	0.1 (0-0.2)	0.52 (0.28 to 0.75)
Islamic Republic of Afghanistan	35.6 (7.9-73.1)	0.6 (0.1-1.3)	46.6 (9.3-108)	0.6 (0.1-1.3)	-0.13 (-0.28 to 0.01)
Islamic Republic of Iran	62.1 (14.4-122.8)	0.3 (0.1-0.7)	176.8 (44.6-344.9)	0.3 (0.1-0.5)	-0.83 (-0.96 to -0.69)
Islamic Republic of Mauritania	4.4 (1.1-8.8)	0.5 (0.1-1.1)	10.5 (2.8-21.7)	0.6 (0.2-1.2)	-0.04 (-0.21 to 0.12)
Islamic Republic of Pakistan	175.6 (45.6-341.8)	0.4 (0.1-0.7)	430.7 (108.3-829.8)	0.4 (0.1-0.8)	0.07 (-0.19 to 0.33)
Jamaica	6.1 (1.6-10.5)	0.3 (0.1-0.6)	12.7 (3.3-23.7)	0.4 (0.1-0.7)	0.01 (-0.6 to 0.63)
Japan	339 (83.2-647.4)	0.2 (0.1-0.4)	1178.4 (304.7-2219.7)	0.2 (0.1-0.4)	-0.32 (-0.46 to -0.17)
Kingdom of Bahrain	0.5 (0.1-1.1)	0.5 (0.1-0.9)	4.3 (1.1-8.8)	0.9 (0.2-1.8)	1.8 (1.51 to 2.09)
Kingdom of Belgium	24.2 (6-48.1)	0.2 (0-0.3)	47.9 (12-94.4)	0.1 (0-0.3)	0.63 (0.27 to 0.99)
Kingdom of Bhutan	0.6 (0.1-1.1)	0.3 (0.1-0.6)	1.3 (0.3-2.6)	0.2 (0.1-0.5)	-0.54 (-0.65 to -0.43)
Kingdom of Cambodia	5.5 (1.2-10.7)	0.2 (0-0.3)	12.7 (3.2-25.9)	0.1 (0-0.3)	-0.93 (-1.24 to -0.61)
Kingdom of Denmark	5.7 (1.4-12)	0.1 (0-0.1)	30.3 (7.7-59.6)	0.2 (0.1-0.4)	4.08 (3.7 to 4.47)
Kingdom of Eswatini	0.8 (0.2-1.5)	0.3 (0.1-0.7)	2.1 (0.5-4.4)	0.4 (0.1-0.9)	1.09 (0.59 to 1.59)
Kingdom of Lesotho	0.9 (0.2-1.9)	0.1 (0-0.3)	2.4 (0.5-5.4)	0.3 (0.1-0.6)	3.25 (2.84 to 3.66)
Kingdom of Morocco	46.7 (10.4-109.9)	0.4 (0.1-0.9)	144.1 (36.8-280.4)	0.5 (0.1-0.9)	0.92 (0.67 to 1.16)
Kingdom of Norway	5.4 (1.3-10.5)	0.1 (0-0.1)	17.5 (4.6-34.6)	0.1 (0-0.3)	2.37 (1.97 to 2.78)
Kingdom of Saudi Arabia	32.8 (8-64.9)	0.7 (0.2-1.3)	185.4 (43.8-371.4)	1.1 (0.3-2.2)	1.37 (1.11 to 1.62)
Kingdom of Spain	96.1 (24.6-186.3)	0.2 (0-0.4)	187.6 (46.8-378.4)	0.1 (0-0.3)	-1.14 (-1.31 to -0.97)
Kingdom of Sweden	9.8 (2.5-19.1)	0.1 (0-0.1)	39.2 (9.8-81.9)	0.1 (0-0.3)	3.24 (3.08 to 3.41)
Kingdom of Thailand	72.8 (18.1-137.4)	0.3 (0.1-0.5)	260.8 (61.6-520.8)	0.2 (0.1-0.5)	-0.47 (-0.57 to -0.37)
Kingdom of Tonga	0.1 (0-0.3)	0.3 (0.1-0.5)	0.3 (0.1-0.6)	0.4 (0.1-0.8)	1.29 (1.15 to 1.43)
Kingdom of the Netherlands	16.5 (3.9-32.5)	0.1 (0-0.2)	59 (13.5-118.8)	0.1 (0-0.3)	1.88 (1.47 to 2.3)
Kyrgyz Republic	1.2 (0.3-2.5)	0 (0-0.1)	5.2 (1.2-10.9)	0.1 (0-0.2)	1.88 (0.98 to 2.78)
Lao People's Democratic Republic	7.6 (1.8-14.4)	0.4 (0.1-0.8)	11.1 (2.4-21.7)	0.3 (0.1-0.6)	-1.44 (-1.55 to -1.33)
Lebanese Republic	8.5 (2.2-16.7)	0.5 (0.1-0.9)	32.5 (8.2-63)	0.5 (0.1-1)	0.58 (0.28 to 0.88)
Malaysia	34.9 (8.8-62.4)	0.4 (0.1-0.7)	106.3 (26.7-207.4)	0.4 (0.1-0.8)	-0.32 (-0.5 to -0.14)
Mongolia	1.7 (0.4-3.5)	0.2 (0-0.3)	3.3 (0.7-6.9)	0.2 (0-0.3)	-0.49 (-0.64 to -0.34)
Montenegro	1.2 (0.3-2.4)	0.2 (0.1-0.4)	2 (0.5-4.3)	0.2 (0.1-0.5)	0.53 (0.18 to 0.89)
New Zealand	4.1 (1.1-8)	0.1 (0-0.2)	13.9 (3.5-27.3)	0.2 (0-0.3)	1.13 (0.58 to 1.68)
North Macedonia	2.2 (0.5-4.3)	0.1 (0-0.3)	4.1 (1-8.1)	0.1 (0-0.3)	0.47 (0.22 to 0.72)
Northern Mariana Islands	0.1 (0-0.2)	1.3 (0.3-2.2)	0.5 (0.1-1)	1.4 (0.4-2.5)	0.36 (0.1 to 0.62)
Palestine	4.1 (1-8.1)	0.6 (0.1-1.2)	9.1 (2.6-18.3)	0.5 (0.1-0.9)	-0.81 (-1 to -0.63)
People's Democratic Republic of Algeria	37.8 (9-79)	0.5 (0.1-1)	152.2 (36.4-294.1)	0.6 (0.1-1.1)	0.91 (0.53 to 1.29)
People's Republic of Bangladesh	70.4 (17.9-136.2)	0.2 (0-0.3)	175.7 (43.6-348.8)	0.1 (0-0.3)	-0.22 (-0.53 to 0.09)
People's Republic of China	1820.5 (465.7-3379.8)	0.3 (0.1-0.5)	4721.8 (1210.1-9005.7)	0.3 (0.1-0.5)	-0.67 (-0.8 to -0.53)
Plurinational State of Bolivia	21.4 (5.4-40.9)	0.8 (0.2-1.5)	66.7 (15.9-121.4)	0.8 (0.2-1.5)	0.3 (0.22 to 0.37)
Portuguese Republic	32.7 (8.8-61)	0.3 (0.1-0.5)	80.5 (20.5-161.1)	0.2 (0.1-0.5)	-0.58 (-1.07 to -0.09)
Principality of Andorra	0.1 (0-0.2)	0.3 (0.1-0.5)	0.3 (0.1-0.6)	0.2 (0-0.3)	-1.03 (-1.26 to -0.81)
Principality of Monaco	0.1 (0-0.2)	0.1 (0-0.3)	0.3 (0.1-0.5)	0.2 (0.1-0.4)	1.38 (1.09 to 1.68)
Puerto Rico	26.3 (6.8-45.5)	0.8 (0.2-1.3)	60.8 (13.8-108.6)	0.8 (0.2-1.3)	0.79 (0.21 to 1.37)
Republic of Albania	2.3 (0.5-4.9)	0.1 (0-0.3)	4.9 (1.3-10.3)	0.1 (0-0.2)	0.16 (-0.1 to 0.41)
Republic of Angola	9.9 (2.4-19.9)	0.3 (0.1-0.7)	28.2 (6.5-60.1)	0.3 (0.1-0.7)	-0.37 (-0.51 to -0.24)
Republic of Armenia	0.2 (0.1-0.5)	0 (0-0)	4.4 (1-9)	0.1 (0-0.2)	7.62 (6.47 to 8.79)
Republic of Austria	10.5 (2.6-21.8)	0.1 (0-0.2)	62.8 (15.3-121.1)	0.3 (0.1-0.5)	5 (4.29 to 5.71)
Republic of Azerbaijan	3 (0.7-6)	0.1 (0-0.1)	9 (2.1-19)	0.1 (0-0.2)	1.7 (1.37 to 2.03)

Republic of Belarus	0.8 (0.2-1.7)	0 (0-0)	3.8 (0.9-8.3)	0 (0-0.1)	4.2 (3.49 to 4.91)
Republic of Benin	5 (1.2-9.8)	0.3 (0.1-0.6)	11.4 (2.9-22.3)	0.3 (0.1-0.5)	-0.24 (-0.32 to -0.16)
Republic of Botswana	0.9 (0.2-1.8)	0.2 (0.1-0.4)	2.7 (0.6-5.8)	0.2 (0.1-0.5)	0.43 (0.23 to 0.62)
Republic of Bulgaria	14.8 (3.7-28.4)	0.2 (0-0.3)	33.5 (8.4-68.6)	0.2 (0.1-0.5)	1.99 (1.6 to 2.38)
Republic of Burundi	7.8 (1.8-15.4)	0.4 (0.1-0.8)	13 (2.8-26.9)	0.4 (0.1-0.8)	-0.66 (-0.8 to -0.52)
Republic of Cabo Verde	0.4 (0.1-0.8)	0.2 (0-0.4)	1.2 (0.3-2.6)	0.3 (0.1-0.6)	1.06 (0.77 to 1.36)
Republic of Cameroon	19.6 (4.9-39.6)	0.6 (0.2-1.2)	56.5 (14-116)	0.6 (0.2-1.2)	-0.08 (-0.27 to 0.11)
Republic of Chad	10.4 (2.6-21)	0.4 (0.1-0.9)	17.6 (4.4-38.3)	0.4 (0.1-0.8)	-0.64 (-0.81 to -0.46)
Republic of Chile	24.9 (6.4-48)	0.3 (0.1-0.5)	67.4 (17.1-131.9)	0.3 (0.1-0.5)	-0.07 (-0.48 to 0.35)
Republic of Colombia	35.1 (8.6-67.6)	0.2 (0.1-0.4)	93.6 (23.5-187.4)	0.2 (0-0.3)	-0.8 (-0.99 to -0.61)
Republic of Costa Rica	4.2 (1.1-8.1)	0.3 (0.1-0.5)	19.8 (5.1-38.7)	0.4 (0.1-0.7)	1.09 (0.63 to 1.55)
Republic of Croatia	7.2 (1.8-14)	0.1 (0-0.3)	15.2 (3.7-30.2)	0.2 (0-0.3)	0.36 (0.03 to 0.69)
Republic of Cuba	23.1 (6.1-39.7)	0.2 (0.1-0.4)	72.4 (19.5-130.1)	0.4 (0.1-0.6)	1.99 (1.67 to 2.32)
Republic of Cyprus	1.9 (0.5-3.7)	0.5 (0.1-0.9)	3.7 (0.9-7.4)	0.3 (0.1-0.5)	-1.94 (-2.22 to -1.65)
Republic of Côte d'Ivoire	12.9 (2.9-26.1)	0.5 (0.1-0.9)	35.3 (8.7-69.9)	0.4 (0.1-0.8)	-0.71 (-0.81 to -0.61)
Republic of Djibouti	0.3 (0.1-0.6)	0.3 (0.1-0.7)	2 (0.5-4.1)	0.5 (0.1-1)	1.11 (0.98 to 1.25)
Republic of Ecuador	30.4 (7.8-53.6)	0.6 (0.2-1.1)	131.2 (30.9-248.7)	0.9 (0.2-1.6)	0.96 (0.08 to 1.84)
Republic of El Salvador	10.6 (2.6-20.4)	0.4 (0.1-0.7)	52.9 (12.9-109.1)	0.8 (0.2-1.7)	2.65 (2.05 to 3.26)
Republic of Equatorial Guinea	1 (0.3-2.1)	0.7 (0.2-1.3)	3.4 (0.8-7.1)	0.8 (0.2-1.7)	0.85 (0.39 to 1.33)
Republic of Estonia	0.7 (0.2-1.5)	0 (0-0.1)	5.5 (1.3-11.3)	0.2 (0-0.3)	4.56 (4.05 to 5.08)
Republic of Fiji	2 (0.5-3.7)	0.7 (0.2-1.3)	8.3 (2.2-16.1)	1.4 (0.4-2.7)	2.03 (1.61 to 2.45)
Republic of Finland	4.3 (1.1-8.2)	0.1 (0-0.1)	12.5 (3.1-25.2)	0.1 (0-0.1)	1.25 (0.99 to 1.52)
Republic of Ghana	19.8 (4.8-39)	0.5 (0.1-0.9)	88.9 (21.9-177.5)	0.7 (0.2-1.4)	1.68 (1.56 to 1.79)
Republic of Guatemala	11.5 (2.8-22.8)	0.4 (0.1-0.9)	45.6 (11.3-91.3)	0.4 (0.1-0.9)	0.59 (0.25 to 0.93)
Republic of Guinea	10.8 (2.7-22.5)	0.4 (0.1-0.8)	17 (4.2-36.6)	0.4 (0.1-0.7)	-0.29 (-0.38 to -0.19)
Republic of Guinea-Bissau	1.4 (0.3-2.9)	0.5 (0.1-0.9)	2 (0.5-4.4)	0.4 (0.1-0.8)	-0.79 (-0.84 to -0.74)
Republic of Guyana	1.4 (0.3-2.6)	0.4 (0.1-0.7)	4.5 (1-8.1)	0.7 (0.2-1.3)	2.91 (2.54 to 3.28)
Republic of Haiti	14.3 (3.1-31.3)	0.5 (0.1-1.2)	26.8 (5.9-65.3)	0.4 (0.1-1)	-0.48 (-0.61 to -0.35)
Republic of Honduras	2.4 (0.6-4.5)	0.1 (0-0.3)	11.6 (2.7-24.2)	0.2 (0-0.4)	1.7 (1.49 to 1.91)
Republic of Iceland	0.2 (0.1-0.4)	0.1 (0-0.1)	0.8 (0.2-1.5)	0.1 (0-0.2)	1.71 (1.5 to 1.91)
Republic of India	669 (168.4-1257.3)	0.2 (0-0.3)	2054.4 (536.2-4028.2)	0.2 (0.1-0.4)	0.45 (0.31 to 0.59)
Republic of Indonesia	139 (35-266.2)	0.2 (0-0.3)	380 (93.7-713.9)	0.2 (0-0.4)	0.25 (0.19 to 0.32)
Republic of Iraq	50 (12.9-96.9)	0.7 (0.2-1.3)	117.1 (28.6-245.5)	0.6 (0.1-1.2)	-0.65 (-1.01 to -0.28)
Republic of Italy	155.4 (39.2-305.3)	0.2 (0-0.4)	421 (110.2-816.3)	0.2 (0.1-0.4)	0.35 (0.21 to 0.48)
Republic of Kazakhstan	6.3 (1.4-12.8)	0 (0-0.1)	18 (4.1-38)	0.1 (0-0.2)	2.21 (1.81 to 2.62)
Republic of Kenya	16.4 (3.9-35.4)	0.3 (0.1-0.5)	70.1 (17.7-144.9)	0.4 (0.1-0.9)	1.78 (1.7 to 1.86)
Republic of Kiribati	0.2 (0.1-0.5)	0.8 (0.2-1.4)	0.6 (0.1-1.2)	1 (0.3-2.1)	0.96 (0.81 to 1.11)
Republic of Korea	39.2 (9.3-77)	0.2 (0-0.4)	121 (28.7-245.7)	0.1 (0-0.3)	-1.4 (-1.54 to -1.25)
Republic of Latvia	0.8 (0.2-1.6)	0 (0-0)	4.1 (0.9-8.6)	0.1 (0-0.2)	4.86 (4.42 to 5.3)
Republic of Liberia	4.8 (1.2-9.4)	0.5 (0.1-1)	9.1 (2.3-19.2)	0.5 (0.1-1)	0.08 (-0.2 to 0.36)
Republic of Lithuania	0.7 (0.2-1.6)	0 (0-0)	4.3 (1-8.9)	0.1 (0-0.1)	3.21 (2.73 to 3.68)
Republic of Madagascar	9.7 (2.1-20.2)	0.2 (0.1-0.5)	19.2 (4.2-40.8)	0.3 (0.1-0.5)	0.14 (0.07 to 0.2)
Republic of Malawi	8.1 (1.8-16.1)	0.3 (0.1-0.6)	20.9 (5.1-42.2)	0.4 (0.1-0.7)	0.68 (0.52 to 0.85)
Republic of Maldives	0.5 (0.1-0.9)	0.7 (0.2-1.4)	0.8 (0.2-1.6)	0.3 (0.1-0.5)	-3.06 (-3.3 to -2.83)
Republic of Mali	13.1 (3.2-25.9)	0.5 (0.1-0.9)	23.7 (5.6-47.8)	0.4 (0.1-0.7)	-0.66 (-0.77 to -0.56)
Republic of Malta	0.8 (0.2-1.7)	0.2 (0.1-0.4)	1.9 (0.5-3.7)	0.2 (0-0.3)	-0.72 (-1.13 to -0.3)
Republic of Mauritius	3.5 (0.9-6.8)	0.5 (0.1-1)	22.9 (5.9-41.6)	1.3 (0.3-2.4)	3.51 (3.02 to 3.99)

Republic of Moldova	0.6 (0.1-1.3)	0 (0-0)	1.9 (0.4-4.2)	0 (0-0.1)	1.75 (1.18 to 2.33)
Republic of Mozambique	9.9 (2.2-20.4)	0.2 (0.1-0.5)	23.3 (5.7-48.3)	0.3 (0.1-0.6)	1.16 (1.02 to 1.29)
Republic of Namibia	1.5 (0.4-3.2)	0.3 (0.1-0.6)	3.5 (0.8-7.6)	0.3 (0.1-0.6)	-0.2 (-0.51 to 0.1)
Republic of Nauru	0 (0-0.1)	0.7 (0.2-1.5)	0.1 (0-0.1)	1.2 (0.3-2.5)	1.67 (1.54 to 1.79)
Republic of Nicaragua	3.9 (1-8)	0.3 (0.1-0.6)	20.7 (5.1-40.6)	0.4 (0.1-0.8)	2.06 (1.7 to 2.43)
Republic of Niue	0 (0-0)	0.7 (0.2-1.3)	0 (0-0.1)	1.5 (0.3-3.1)	2.73 (2.62 to 2.84)
Republic of Palau	0.1 (0-0.1)	0.7 (0.2-1.3)	0.2 (0-0.4)	1.3 (0.3-2.5)	2.21 (2.05 to 2.38)
Republic of Panama	2.8 (0.7-5.3)	0.2 (0-0.4)	14.1 (3.3-28.1)	0.3 (0.1-0.6)	1.56 (1.09 to 2.04)
Republic of Paraguay	8.4 (2.1-15.8)	0.4 (0.1-0.8)	22.1 (5.8-42.8)	0.4 (0.1-0.8)	0.24 (0.08 to 0.4)
Republic of Peru	56 (13.6-110.3)	0.5 (0.1-1)	162.8 (41.4-326.9)	0.5 (0.1-1)	-0.54 (-0.77 to -0.31)
Republic of Poland	99.9 (24.6-194.6)	0.2 (0.1-0.5)	81.5 (20.4-159.6)	0.1 (0-0.2)	-3.19 (-3.82 to -2.55)
Republic of Rwanda	6.5 (1.5-13.2)	0.3 (0.1-0.6)	11.2 (2.5-22.9)	0.3 (0.1-0.5)	-1.54 (-1.89 to -1.2)
Republic of San Marino	0 (0-0.1)	0.1 (0-0.2)	0.1 (0-0.2)	0.1 (0-0.1)	-0.32 (-0.78 to 0.14)
Republic of Senegal	16.2 (4-32.7)	0.6 (0.2-1.2)	31.6 (7.8-65.9)	0.5 (0.1-1)	-1.14 (-1.33 to -0.95)
Republic of Serbia	18.7 (4.2-37.9)	0.2 (0-0.4)	27.4 (6.7-53.6)	0.2 (0-0.3)	-0.82 (-1.11 to -0.54)
Republic of Seychelles	0.2 (0.1-0.4)	0.4 (0.1-0.7)	0.6 (0.1-1.1)	0.5 (0.1-1)	1.09 (0.92 to 1.25)
Republic of Sierra Leone	5.4 (1.4-10.4)	0.3 (0.1-0.6)	7.8 (1.8-15.3)	0.3 (0.1-0.5)	-0.79 (-0.9 to -0.68)
Republic of Singapore	4 (1.1-7.6)	0.2 (0.1-0.4)	14.7 (3.6-28.4)	0.2 (0-0.3)	0.81 (0.26 to 1.37)
Republic of Slovenia	2.1 (0.5-4.3)	0.1 (0-0.2)	5.1 (1.3-10.4)	0.1 (0-0.2)	0.98 (0.66 to 1.3)
Republic of South Africa	31.7 (7.2-61.5)	0.2 (0-0.3)	104.2 (25.5-209.4)	0.3 (0.1-0.5)	1.63 (1.34 to 1.93)
Republic of South Sudan	8.6 (1.9-17.8)	0.4 (0.1-0.8)	15.9 (4.1-33.3)	0.6 (0.1-1.1)	0.92 (0.78 to 1.06)
Republic of Sudan	26.4 (5.7-58.8)	0.3 (0.1-0.7)	72.4 (17.5-145.2)	0.4 (0.1-0.8)	0.74 (0.49 to 1)
Republic of Suriname	1.3 (0.3-2.3)	0.5 (0.1-0.9)	3.7 (1-7.1)	0.6 (0.2-1.2)	0.87 (0.65 to 1.1)
Republic of Tajikistan	0.4 (0.1-0.7)	0 (0-0)	0.9 (0.2-2)	0 (0-0)	0.22 (-0.27 to 0.71)
Republic of Trinidad and Tobago	2.7 (0.7-4.7)	0.4 (0.1-0.6)	12.4 (3.1-22.8)	0.6 (0.2-1.2)	2.63 (2.26 to 3.01)
Republic of Tunisia	10.9 (2.7-23.5)	0.3 (0.1-0.6)	43.3 (10.5-89.9)	0.4 (0.1-0.8)	1.14 (1.04 to 1.23)
Republic of Turkey	163.3 (41.6-321.7)	0.6 (0.1-1.1)	396.7 (103.2-808.6)	0.5 (0.1-1)	-0.44 (-0.73 to -0.16)
Republic of Uganda	14 (3.3-29.7)	0.3 (0.1-0.6)	35.2 (8.2-70.9)	0.3 (0.1-0.6)	0.15 (0.02 to 0.28)
Republic of Uzbekistan	5.9 (1.1-13.8)	0.1 (0-0.1)	32.6 (7.4-65.1)	0.1 (0-0.3)	1.75 (0.92 to 2.58)
Republic of Vanuatu	0.3 (0.1-0.8)	0.7 (0.2-1.6)	0.9 (0.2-1.8)	0.7 (0.2-1.3)	-0.34 (-0.42 to -0.27)
Republic of Yemen	14.1 (3-32.3)	0.4 (0.1-0.9)	30.5 (7.8-67.8)	0.3 (0.1-0.6)	-1.25 (-1.42 to -1.08)
Republic of Zambia	9.1 (2.1-18)	0.4 (0.1-0.8)	25.2 (6-50.6)	0.5 (0.1-0.9)	-0.01 (-0.18 to 0.17)
Republic of Zimbabwe	9.8 (2.3-19.9)	0.3 (0.1-0.6)	22 (5.2-48.6)	0.4 (0.1-0.8)	0.86 (0.38 to 1.34)
Republic of the Congo	5 (1.2-10.4)	0.6 (0.2-1.2)	10.5 (2.6-22.3)	0.5 (0.1-1)	-0.73 (-0.86 to -0.6)
Republic of the Gambia	1 (0.2-1.9)	0.4 (0.1-0.7)	3.3 (0.8-6.6)	0.4 (0.1-0.8)	0.06 (-0.06 to 0.18)
Republic of the Marshall Islands	0.1 (0-0.2)	0.6 (0.1-1.4)	0.4 (0.1-1.1)	1.3 (0.2-4)	2.78 (2.63 to 2.93)
Republic of the Niger	7.6 (1.9-16.3)	0.3 (0.1-0.7)	18.3 (4.3-41.9)	0.3 (0.1-0.6)	-0.72 (-0.78 to -0.66)
Republic of the Philippines	51.9 (12.3-103.8)	0.2 (0.1-0.5)	219 (54.8-428.5)	0.3 (0.1-0.6)	1.17 (1.03 to 1.31)
Republic of the Union of Myanmar	50.9 (13.2-93.6)	0.2 (0.1-0.5)	82.9 (20.9-160.7)	0.2 (0-0.4)	-0.99 (-1.07 to -0.9)
Romania	38.9 (8.9-80.2)	0.2 (0-0.3)	60.6 (14.1-113.5)	0.2 (0-0.3)	0.42 (-0.11 to 0.96)
Russian Federation	71.7 (16.6-150.7)	0 (0-0.1)	182.6 (46.4-361.9)	0.1 (0-0.1)	1.69 (1.22 to 2.16)
Saint Kitts and Nevis	0.3 (0.1-0.5)	0.8 (0.2-1.4)	0.5 (0.1-0.9)	0.9 (0.2-1.6)	1.42 (1.1 to 1.74)
Saint Lucia	0.4 (0.1-0.8)	0.6 (0.1-1)	1.4 (0.4-2.6)	0.6 (0.2-1.1)	0.53 (0.24 to 0.82)
Saint Vincent and the Grenadines	0.3 (0.1-0.5)	0.4 (0.1-0.7)	0.8 (0.2-1.4)	0.6 (0.1-1.1)	1.89 (1.55 to 2.23)
Slovak Republic	9.7 (2.4-19.3)	0.2 (0-0.3)	13.2 (3.1-27)	0.1 (0-0.3)	-0.46 (-0.58 to -0.34)
Socialist Republic of Viet Nam	65.9 (15.4-127.1)	0.2 (0-0.4)	149 (35.6-299.6)	0.2 (0-0.4)	-0.07 (-0.22 to 0.08)
Solomon Islands	0.7 (0.1-1.6)	0.7 (0.2-1.4)	1.3 (0.3-2.4)	0.5 (0.1-0.8)	-1.69 (-1.93 to -1.46)

State of Eritrea	1.8 (0.4-3.8)	0.2 (0.1-0.5)	5.4 (1.2-11.3)	0.3 (0.1-0.6)	0.56 (0.46 to 0.66)
State of Israel	17.3 (4.2-33.5)	0.4 (0.1-0.8)	36.2 (9.5-73.5)	0.2 (0.1-0.5)	-1.17 (-1.65 to -0.68)
State of Kuwait	1.9 (0.5-3.7)	0.3 (0.1-0.7)	6.4 (1.5-12.6)	0.3 (0.1-0.6)	-0.25 (-0.47 to -0.03)
State of Libya	5.2 (1.3-10.4)	0.3 (0.1-0.6)	22.3 (6-46.2)	0.5 (0.1-1)	2.31 (2.04 to 2.59)
State of Qatar	0.3 (0.1-0.7)	0.5 (0.1-1)	4.2 (1-8.3)	0.9 (0.2-1.8)	2.16 (1.47 to 2.86)
Sultanate of Oman	2.2 (0.5-4.2)	0.4 (0.1-0.8)	9 (2.3-17.3)	0.6 (0.2-1.1)	1.85 (1.36 to 2.34)
Swiss Confederation	11.4 (2.8-23)	0.1 (0-0.2)	30.5 (7.9-60.9)	0.1 (0-0.2)	1.45 (1.08 to 1.83)
Syrian Arab Republic	27.1 (6.6-53)	0.6 (0.2-1.2)	59.4 (15.5-122)	0.6 (0.2-1.1)	-0.8 (-1.09 to -0.5)
Taiwan (Province of China)	79.5 (20.3-142.1)	0.7 (0.2-1.3)	194.4 (49.4-373.4)	0.4 (0.1-0.8)	-1.46 (-1.78 to -1.13)
Togolese Republic	3.4 (0.9-6.7)	0.4 (0.1-0.8)	10.5 (2.7-21.6)	0.4 (0.1-0.8)	-0.07 (-0.16 to 0.01)
Tokelau	0 (0-0)	0.5 (0.1-1)	0 (0-0)	0.9 (0.2-1.6)	1.99 (1.89 to 2.1)
Turkmenistan	1.6 (0.4-3.3)	0.1 (0-0.2)	7.7 (1.9-15.5)	0.2 (0-0.4)	2.45 (1.85 to 3.06)
Tuvalu	0 (0-0.1)	0.7 (0.2-1.4)	0.1 (0-0.2)	0.9 (0.2-1.8)	1.24 (1.18 to 1.31)
Ukraine	0.8 (0.2-1.6)	0 (0-0)	15.1 (3.3-34.7)	0 (0-0)	13.36 (11.43 to 15.31)
Union of the Comoros	0.4 (0.1-0.8)	0.3 (0.1-0.5)	1.1 (0.3-2.2)	0.3 (0.1-0.6)	0.03 (-0.07 to 0.13)
United Arab Emirates	1.3 (0.3-2.6)	0.3 (0.1-0.7)	13.4 (3.2-25.9)	0.7 (0.2-1.4)	5.43 (4.55 to 6.31)
United Kingdom of Great Britain and Northern Ireland	45.3 (11.5-90.6)	0 (0-0.1)	89.2 (21.4-172)	0.1 (0-0.1)	1.39 (1.07 to 1.7)
United Mexican States	168.9 (42.9-314.1)	0.5 (0.1-0.9)	816.8 (212.2-1553.4)	0.7 (0.2-1.3)	1.69 (1.08 to 2.31)
United Republic of Tanzania	24.5 (5.9-49.5)	0.3 (0.1-0.6)	72 (16.6-148.3)	0.3 (0.1-0.7)	0.37 (0.3 to 0.44)
United States Virgin Islands	0.3 (0.1-0.5)	0.3 (0.1-0.6)	0.6 (0.2-1.2)	0.4 (0.1-0.7)	0.76 (0.54 to 0.97)
United States of America	550.8 (149.2-1056.4)	0.2 (0-0.3)	2380.1 (622.2-4371.8)	0.4 (0.1-0.7)	2.84 (2.63 to 3.05)

TableS2 The DALYs burden of CKD attributable to diet low in whole grains in 204 countries and territories, 1990 - 2021.

location	1990		2021		EAPC_95%CI
	Number(95% UI)	ASR(95% UI)	Number(95% UI)	ASR(95% UI)	
American Samoa	3.8 (1-7.2)	17 (4.4-31.4)	21 (5.3-40.1)	45.2 (11.4-85.1)	3.44 (3.19 to 3.69)
Antigua and Barbuda	6.9 (1.6-12.2)	13.3 (3.1-23.7)	17.5 (4.4-31.1)	16.5 (4.2-29.3)	1.33 (1.06 to 1.6)
Arab Republic of Egypt	3189.4 (751.5-6358.7)	14.1 (3.3-28.1)	8719.1 (2283.4-18031.8)	14.9 (4-30)	0.1 (-0.02 to 0.22)
Argentine Republic	4326.5 (1057.9-8166.6)	13.7 (3.3-25.8)	5569.4 (1390-10319.1)	9.9 (2.5-18.5)	-0.75 (-1.05 to -0.45)
Australia	490.5 (116.4-955.3)	2.6 (0.6-5)	1517.1 (361.7-2911.5)	3.1 (0.8-6.2)	1.02 (0.82 to 1.23)
Barbados	32.8 (8.5-58.6)	12.2 (3.2-21.7)	66.9 (16.3-124.2)	13.7 (3.3-25.7)	0.97 (0.71 to 1.23)
Belize	10.6 (2.6-19.7)	11.2 (2.8-20.6)	53.3 (12.8-96.6)	16.3 (3.9-29.1)	1.51 (1.11 to 1.92)
Bermuda	6.9 (1.7-12.6)	11.2 (2.7-20.3)	12.1 (3.1-22.1)	9.3 (2.3-17)	-0.09 (-0.37 to 0.19)
Bolivarian Republic of Venezuela	631.7 (148.7-1305.1)	6.3 (1.5-12.7)	3100 (706.2-6222.7)	10.2 (2.3-20.5)	1.26 (0.87 to 1.64)
Bosnia and Herzegovina	127.2 (30.2-273)	3.2 (0.8-7.1)	155.5 (38.8-324.4)	2.6 (0.6-5.4)	-0.76 (-1.07 to -0.46)
Brunei Darussalam	7.5 (1.7-14.4)	7.7 (1.9-14.5)	22 (5.4-43.8)	7.1 (1.8-13.7)	0.17 (-0.01 to 0.35)
Burkina Faso	299.6 (69-601.3)	7.7 (1.8-15.2)	662.8 (145.6-1330.4)	7.5 (1.7-14.9)	-0.08 (-0.14 to -0.03)
Canada	628.5 (159.6-1225.3)	1.9 (0.5-3.8)	1493.5 (350.7-2886.2)	2 (0.5-4)	0.5 (0.25 to 0.75)
Central African Republic	232.3 (51.6-478.8)	18.9 (4.4-38.4)	407.3 (94.8-868.3)	15.5 (3.8-32.4)	-0.71 (-0.77 to -0.66)
Commonwealth of Dominica	8.1 (2-14.3)	14.2 (3.5-25.2)	15.7 (4-29.2)	19.1 (5-35.6)	1.26 (1.15 to 1.36)
Commonwealth of the Bahamas	22.5 (5.5-41.4)	13.7 (3.4-24.7)	68.8 (18.6-125.3)	16.1 (4.3-29.3)	1.16 (0.94 to 1.37)
Cook Islands	1.2 (0.3-2.2)	9.8 (2.7-17.6)	3.4 (0.9-6.4)	13.4 (3.7-24.9)	1.12 (1.04 to 1.2)
Czech Republic	412 (98.1-825.8)	3.1 (0.7-6.2)	398.4 (98.6-795.6)	2 (0.5-4)	-1.46 (-1.57 to -1.34)
Democratic People's Republic of Korea	1265.9 (291.4-2362.9)	8.3 (1.9-15.6)	2331.7 (550.4-4667.9)	7.1 (1.7-14.2)	-0.38 (-0.5 to -0.26)
Democratic Republic of Sao Tome and Principe	6.5 (1.5-13)	10.8 (2.5-21.8)	14.1 (3.3-29.8)	13.2 (3.3-27.3)	0.63 (0.53 to 0.73)
Democratic Republic of Timor-Leste	13.7 (3-27.3)	5.3 (1.3-10.3)	36.8 (8.1-76)	4.4 (1-9)	-0.55 (-0.83 to -0.26)

Democratic Republic of the Congo	3161.3 (759.3-6165.2)	20 (5-39)	6882.9 (1773.4-14376.3)	17.1 (4.5-35)	-0.75 (-0.87 to -0.64)
Democratic Socialist Republic of Sri Lanka	529.2 (124.3-1007.8)	5 (1.2-9.3)	1115.9 (275.7-2304.4)	4.2 (1-8.7)	-0.69 (-0.96 to -0.41)
Dominican Republic	294.6 (66.2-570.5)	8.3 (1.9-15.9)	1008 (270.2-1907.7)	9.9 (2.7-18.7)	1.37 (1.14 to 1.59)
Eastern Republic of Uruguay	165.5 (37.7-316.3)	4.3 (1-8.2)	260.6 (65.5-538.1)	4.4 (1.1-9.3)	0.45 (0.2 to 0.71)
Federal Democratic Republic of Ethiopia	3815.5 (929.2-7254.2)	20.7 (5.1-39.4)	4646.3 (1086.7-9077)	11.9 (2.8-23.2)	-2.28 (-2.44 to -2.12)
Federal Democratic Republic of Nepal	545.4 (121.2-1069.5)	5.9 (1.4-11.4)	1551 (334.5-3137.3)	6.6 (1.5-13.1)	0.25 (0.04 to 0.46)
Federal Republic of Germany	3376.2 (822.1-6697.5)	2.6 (0.6-5.2)	5945.4 (1424-11581.1)	2.7 (0.6-5.3)	0.49 (0.25 to 0.73)
Federal Republic of Nigeria	3836.9 (854.6-7578.3)	9 (2.1-17.4)	7671 (1825.8-15451.2)	8 (1.9-15.8)	-0.66 (-0.82 to -0.5)
Federal Republic of Somalia	203.1 (42-434.4)	9.6 (2.1-20.5)	559.6 (132.4-1217.8)	9.9 (2.5-21)	0.15 (0.02 to 0.29)
Federated States of Micronesia	10.6 (2.6-21.8)	22.2 (5.7-46.2)	21.7 (5.6-43.8)	30.1 (7.6-59)	1.2 (1.06 to 1.33)
Federative Republic of Brazil	8487 (2021.5-15851.9)	9.3 (2.2-17.1)	22890.7 (5876.5-42576.6)	9.1 (2.3-16.9)	-0.22 (-0.38 to -0.05)
French Republic	2681.1 (632.3-5238)	3.1 (0.7-6.1)	5081.8 (1279.6-10078.1)	3 (0.7-5.9)	-0.05 (-0.18 to 0.08)
Gabonese Republic	90.8 (19.5-187.8)	16.1 (3.5-33.1)	229.5 (54.2-465.1)	21.2 (5.3-43.3)	0.76 (0.53 to 0.98)
Georgia	157.5 (34.7-332.6)	2.6 (0.6-5.4)	223.2 (51.1-457.1)	3.9 (0.9-8)	1.66 (1.33 to 2.01)
Grand Duchy of Luxembourg	23.9 (5.8-47.8)	4.5 (1.1-8.9)	29.6 (8.1-58.5)	2.6 (0.7-5.3)	-1.73 (-1.95 to -1.5)
Greenland	1 (0.2-1.9)	3.3 (0.8-6.3)	1.9 (0.5-3.6)	3 (0.7-5.7)	0.06 (-0.05 to 0.17)
Grenada	10.7 (2.7-19)	15.9 (3.9-28.4)	25.3 (6.2-45.1)	21.9 (5.4-38.8)	1.61 (1.41 to 1.82)
Guam	9.1 (2.3-17.2)	12.7 (3.4-23.8)	24.9 (6.7-47.8)	11.9 (3.2-23.1)	0.58 (0.3 to 0.87)
Hashemite Kingdom of Jordan	154.9 (36.9-297)	11.2 (2.7-21.6)	954.8 (229.2-1830.6)	13 (3.2-25.2)	0.5 (0.21 to 0.79)
Hellenic Republic	1220.5 (312.4-2492)	8.3 (2.2-16.8)	2214.2 (538.4-4407.4)	7.6 (1.8-15.2)	-0.6 (-1.23 to 0.03)
Hungary	358.9 (88.1-698.8)	2.6 (0.6-5.1)	476.1 (117.5-923.9)	2.5 (0.6-5)	0.57 (0.27 to 0.87)
Independent State of Papua New Guinea	122.7 (29.1-236.3)	6.4 (1.7-12.2)	305.5 (69.9-606.2)	5.5 (1.3-10.9)	-0.52 (-0.63 to -0.42)
Independent State of Samoa	13.3 (2.9-25.4)	15.4 (3.4-29.6)	35 (9-68.6)	24.5 (6.4-47.5)	1.73 (1.61 to 1.86)
Ireland	148.7 (34.9-294)	3.8 (0.9-7.4)	264.8 (68.7-521.4)	3.3 (0.8-6.5)	-0.09 (-0.18 to 0.01)
Islamic Republic of Afghanistan	981.7 (215.8-2030)	14.2 (3.2-29.6)	1439.8 (281.6-3332.2)	13.2 (2.7-30.5)	-0.2 (-0.32 to -0.08)
Islamic Republic of Iran	1832.2 (415.8-3628.5)	7.5 (1.7-14.9)	4662.2 (1175.2-9052.9)	6 (1.5-11.6)	-0.71 (-0.81 to -0.6)
Islamic Republic of Mauritania	109.6 (26.7-222.6)	11.7 (2.9-23.6)	255.5 (64.4-539)	12.5 (3.3-25.6)	-0.12 (-0.25 to 0.02)
Islamic Republic of Pakistan	4996.1 (1266.8-9859.3)	8.9 (2.3-17.2)	14420.3 (3419.6-28428.2)	10.6 (2.6-20.7)	0.29 (0.06 to 0.52)
Jamaica	128.6 (34.2-230.1)	7.2 (1.9-12.9)	306.4 (73.9-580.3)	9.8 (2.3-18.5)	0.55 (-0.01 to 1.12)
Japan	7370.3 (1685.5-14056.1)	4.6 (1-8.6)	18234.8 (4634-35354.3)	4.1 (1-7.9)	-0.13 (-0.28 to 0.03)
Kingdom of Bahrain	18.3 (4.3-36.3)	9.9 (2.4-19.4)	120.8 (30.8-245)	15.6 (4-32.1)	1.24 (1.04 to 1.44)
Kingdom of Belgium	606.6 (138.4-1193.8)	3.9 (0.9-7.7)	910.5 (228.5-1796.4)	3.5 (0.9-6.9)	0.24 (0 to 0.48)
Kingdom of Bhutan	18.8 (4.2-38.7)	7.4 (1.8-15.3)	37.7 (8.8-75.2)	6 (1.4-11.7)	-0.69 (-0.79 to -0.59)
Kingdom of Cambodia	156.8 (33.7-305.7)	3.8 (0.8-7.4)	361.6 (88.4-744.6)	3 (0.7-6.2)	-0.94 (-1.19 to -0.69)
Kingdom of Denmark	171.9 (42.1-356.1)	2 (0.5-4.3)	525.4 (127.4-1022.4)	4 (1-8)	2.29 (2.05 to 2.54)
Kingdom of Eswatini	24.1 (5.7-47.8)	8.5 (2-16.6)	68.9 (16-142.4)	11.4 (2.7-23.2)	1.17 (0.7 to 1.64)
Kingdom of Lesotho	28.8 (6.8-58.8)	3.6 (0.9-7.3)	75.4 (17-164.2)	7 (1.6-15)	2.77 (2.45 to 3.08)
Kingdom of Morocco	1202.8 (276.8-2527.6)	8.5 (1.9-18.6)	3690.7 (911.9-7119.2)	10.7 (2.7-20.4)	0.97 (0.8 to 1.14)
Kingdom of Norway	183.9 (49-366.1)	2.7 (0.7-5.3)	349.3 (90.2-690.2)	3.2 (0.8-6.3)	0.7 (0.52 to 0.88)
Kingdom of Saudi Arabia	948.5 (219.4-1909.8)	15.2 (3.7-29.9)	6373.4 (1477.1-12796.8)	25.6 (6.3-49.7)	1.28 (1.03 to 1.54)
Kingdom of Spain	2123.7 (544.3-4181.3)	4 (1-7.9)	3027.9 (759.4-6135.3)	2.7 (0.7-5.3)	-1.24 (-1.4 to -1.07)
Kingdom of Sweden	291.4 (72.7-595.3)	1.9 (0.5-3.8)	650 (157.5-1332.1)	2.6 (0.6-5.3)	1.59 (1.46 to 1.72)
Kingdom of Thailand	2054.5 (488.3-3982.1)	6 (1.4-11.7)	6303.1 (1452.8-12701.5)	5.8 (1.3-11.7)	-0.31 (-0.38 to -0.23)
Kingdom of Tonga	4.5 (1.1-8.5)	7.9 (1.9-15.1)	8.6 (2.2-16.2)	10.7 (2.8-20.1)	0.69 (0.56 to 0.83)
Kingdom of the Netherlands	454.9 (103.5-906.6)	2.3 (0.5-4.5)	1076.2 (251-2135.3)	2.9 (0.7-5.8)	0.79 (0.6 to 0.98)
Kyrgyz Republic	114.7 (24.5-233.5)	3.7 (0.8-7.7)	260.2 (59.5-530.7)	5 (1.1-10.1)	0.29 (-0.17 to 0.75)
Lao People's Democratic Republic	220.4 (50.5-425.9)	10.5 (2.4-20.1)	317.8 (66.3-624.5)	6.7 (1.4-13.1)	-1.6 (-1.69 to -1.52)

Lebanese Republic	221.6 (54-442.7)	10.3 (2.6-20.1)	620.1 (160.1-1216.9)	10 (2.6-19.7)	0.12 (-0.18 to 0.42)
Malaysia	998.5 (248.1-1890.4)	10.8 (2.7-19.8)	2985.6 (702.1-5737.8)	10.4 (2.5-20)	-0.35 (-0.52 to -0.19)
Mongolia	71.3 (16.1-146)	6.5 (1.5-13.2)	153.2 (34.3-323.7)	6.1 (1.4-12.6)	-0.45 (-0.55 to -0.34)
Montenegro	35.1 (8.6-68.5)	5.7 (1.4-11.1)	50 (12.9-101.4)	5.5 (1.4-11.1)	0.02 (-0.14 to 0.18)
New Zealand	110.4 (27.7-223.4)	2.9 (0.7-5.8)	322.8 (79.6-628.7)	3.9 (0.9-7.5)	0.98 (0.53 to 1.43)
North Macedonia	84.1 (19.9-168.8)	4.5 (1.1-9)	133.2 (32.1-261.7)	4.3 (1-8.5)	-0.01 (-0.15 to 0.14)
Northern Mariana Islands	4.5 (1.2-8.1)	26.1 (6.8-47)	14.1 (3.8-27.1)	28.4 (7.7-53.3)	0.43 (0.16 to 0.7)
Palestine	96.6 (23-193.1)	11.7 (2.8-23.2)	245.5 (63.5-509.9)	9.7 (2.7-19.6)	-0.75 (-0.89 to -0.61)
People's Democratic Republic of Algeria	1079.5 (248-2250.3)	9.7 (2.3-20.3)	3744.9 (889-7460.4)	11 (2.7-21.3)	0.54 (0.3 to 0.79)
People's Republic of Bangladesh	2277.1 (537.7-4527.1)	4.7 (1.1-9)	5588.4 (1312.4-11209.3)	4.1 (1-8.1)	-0.08 (-0.37 to 0.21)
People's Republic of China	52890.3 (12885.2-100882.8)	6.8 (1.7-12.8)	114616 (28230.6-226895.3)	5.6 (1.4-11.1)	-0.66 (-0.82 to -0.51)
Plurinational State of Bolivia	535.6 (128.4-1025.3)	17.1 (4.2-32.5)	1637.1 (381.1-3011.4)	18 (4.2-32.7)	0.24 (0.17 to 0.3)
Portuguese Republic	746.2 (198.2-1412.5)	5.7 (1.5-10.8)	1231.7 (306.7-2399.8)	4.4 (1.1-8.5)	-0.91 (-1.31 to -0.5)
Principality of Andorra	2.6 (0.6-5.3)	5.2 (1.2-10.2)	5.9 (1.5-11.5)	3.6 (0.9-7.1)	-0.89 (-1.04 to -0.75)
Principality of Monaco	2.8 (0.7-5.4)	3.8 (1-7.1)	4.7 (1.2-9.2)	4.4 (1.1-8.7)	0.64 (0.48 to 0.8)
Puerto Rico	639.4 (167.3-1118)	18.2 (4.7-31.9)	1169.8 (266-2076.4)	17.8 (4.1-32.4)	0.72 (0.24 to 1.2)
Republic of Albania	70.2 (16-146.4)	3.6 (0.8-7.3)	131.4 (32.8-270.9)	3.1 (0.8-6.4)	-0.01 (-0.19 to 0.17)
Republic of Angola	318.5 (74.6-647.5)	8.3 (2.1-16.6)	905.9 (197.2-1912.7)	7.6 (1.8-15.9)	-0.51 (-0.64 to -0.38)
Republic of Armenia	53.6 (11.9-110.8)	2 (0.4-4)	168.1 (37.4-343.6)	4 (0.9-8.3)	2.51 (2.26 to 2.76)
Republic of Austria	281 (66.1-585.2)	2.3 (0.5-4.7)	950 (222.6-1840.3)	4.4 (1-8.5)	2.99 (2.54 to 3.45)
Republic of Azerbaijan	187.7 (42.6-378.8)	3.6 (0.8-7.3)	450.8 (103-928)	4.1 (1-8.5)	0.5 (0.34 to 0.66)
Republic of Belarus	180.3 (39.8-393.9)	1.4 (0.3-3.2)	301.2 (68.2-635.7)	1.9 (0.4-4.1)	0.88 (0.74 to 1.01)
Republic of Benin	118.6 (27.7-229.5)	6.3 (1.5-12.3)	307.1 (69.3-612.5)	6.1 (1.5-12)	-0.22 (-0.3 to -0.14)
Republic of Botswana	29.6 (6.9-59.6)	5.6 (1.3-11.2)	89.9 (20.5-186.8)	6.1 (1.4-12.6)	0.22 (0.07 to 0.38)
Republic of Bulgaria	553.6 (141.2-1067.3)	4.9 (1.3-9.6)	867.7 (215.2-1759)	6.8 (1.6-13.4)	1.35 (1.09 to 1.6)
Republic of Burundi	183.6 (42.4-364.4)	8.4 (2-16.7)	319.8 (66.6-648)	7.3 (1.6-15.1)	-0.85 (-1 to -0.7)
Republic of Cabo Verde	12.6 (2.9-25.4)	5.5 (1.3-11.2)	35.8 (8.3-72.4)	7.4 (1.7-14.8)	0.71 (0.54 to 0.88)
Republic of Cameroon	512.6 (118.2-1050.8)	12.5 (3-25.4)	1698.7 (411.6-3542.8)	13.2 (3.4-26.9)	0.19 (0.02 to 0.36)
Republic of Chad	272.1 (66-543.8)	10.1 (2.4-20.4)	546.4 (132.1-1155.8)	9.2 (2.3-19)	-0.48 (-0.64 to -0.31)
Republic of Chile	642.1 (164.6-1242.2)	6.5 (1.7-12.7)	1347.1 (338.4-2616)	5.3 (1.3-10.3)	-0.51 (-0.89 to -0.14)
Republic of Colombia	1057.4 (250.9-2160)	5.9 (1.5-11.8)	2474.8 (630.9-5052.3)	4.4 (1.1-9)	-0.79 (-0.96 to -0.62)
Republic of Costa Rica	132.1 (31.8-265.1)	7.4 (1.8-14.8)	531.9 (131.6-1062.9)	9.6 (2.4-19.1)	0.87 (0.51 to 1.24)
Republic of Croatia	222 (54.7-427)	3.8 (0.9-7.4)	320.2 (75.8-619.8)	3.7 (0.9-7.1)	-0.22 (-0.4 to -0.04)
Republic of Cuba	682.1 (175.2-1192.2)	6.7 (1.7-11.7)	1707.8 (465.3-3084)	9 (2.5-16.2)	1.44 (1.19 to 1.7)
Republic of Cyprus	39.4 (10-77.5)	7.3 (1.8-13.8)	73.3 (18.5-142.9)	4.4 (1.1-8.5)	-1.69 (-1.87 to -1.5)
Republic of Côte d'Ivoire	406.2 (92.8-831.2)	10.5 (2.5-20.7)	1155.9 (267.5-2426.6)	9.5 (2.4-18.6)	-0.54 (-0.65 to -0.43)
Republic of Djibouti	7.7 (1.7-15.5)	6.5 (1.5-13.5)	51 (12-106.2)	9 (2.2-18.6)	1 (0.85 to 1.14)
Republic of Ecuador	736 (187.5-1334.7)	14.1 (3.6-25.3)	3090 (706.4-6114.9)	19 (4.4-37.2)	0.95 (0.14 to 1.76)
Republic of El Salvador	293.6 (69.4-566.4)	9.6 (2.3-18.4)	1312.6 (297.9-2747.5)	21.2 (4.8-44.3)	2.72 (2.18 to 3.27)
Republic of Equatorial Guinea	31.2 (7.4-63.2)	16.4 (4-32.5)	114.9 (28.6-231)	18.8 (4.9-37.6)	0.74 (0.29 to 1.19)
Republic of Estonia	38.9 (9.7-84.6)	2 (0.5-4.2)	113.6 (26.8-229.6)	4 (0.9-8.2)	2.1 (1.81 to 2.38)
Republic of Fiji	60.8 (16.4-114)	16.3 (4.4-30.5)	211.5 (54.9-409.6)	29.4 (7.7-57.1)	1.57 (1.26 to 1.89)
Republic of Finland	146.5 (36.1-293)	2.1 (0.5-4.2)	253.1 (61-508.2)	1.9 (0.5-3.8)	0.24 (0.01 to 0.46)
Republic of Ghana	472.1 (107.4-944)	8.9 (2.1-17.1)	2259.8 (527-4599.2)	14.3 (3.5-28.3)	1.88 (1.74 to 2.02)
Republic of Guatemala	333.4 (78.5-694)	9.9 (2.4-19.9)	1324.9 (327.5-2738)	11.5 (2.8-23.6)	1.03 (0.69 to 1.38)
Republic of Guinea	295.8 (70.5-616)	9.2 (2.3-19)	515.2 (127.9-1087.2)	8.6 (2.2-18.2)	-0.18 (-0.24 to -0.11)
Republic of Guinea-Bissau	40.5 (9.2-82.4)	10.5 (2.5-21.2)	68.4 (15.2-147.9)	8.7 (2.1-18.6)	-0.68 (-0.73 to -0.64)

Republic of Guyana	43.3 (10.3-79.3)	10.6 (2.5-19.3)	129.9 (30.1-240.4)	19 (4.5-34.9)	2.91 (2.57 to 3.25)
Republic of Haiti	431.3 (94.1-905.2)	12.9 (2.9-27.6)	853.8 (195.8-1999.3)	10.7 (2.4-25.2)	-0.35 (-0.47 to -0.22)
Republic of Honduras	90.5 (21.9-180.6)	4.3 (1-8.4)	365.6 (81.6-769.1)	5.6 (1.3-11.4)	1.06 (0.93 to 1.19)
Republic of Iceland	7.4 (1.8-14.7)	2.5 (0.6-5)	16.2 (4-32.7)	2.6 (0.7-5.3)	0.32 (0.21 to 0.43)
Republic of India	24262.5 (6059.8-46931.6)	5.1 (1.3-10)	68501.9 (16880.8-139740.1)	5.6 (1.4-11.5)	0.44 (0.37 to 0.51)
Republic of Indonesia	4372.9 (1054.6-8533.8)	4.5 (1.1-8.9)	11886.5 (2855-22498.5)	4.8 (1.2-9.4)	0.21 (0.17 to 0.25)
Republic of Iraq	1410.1 (346.4-2653.9)	16.6 (4.2-31.6)	3498.8 (845.7-7349.8)	13.5 (3.3-27.8)	-0.84 (-1.11 to -0.57)
Republic of Italy	3698.1 (915.6-7353.6)	4.2 (1-8.5)	6567.4 (1710.5-12815.7)	3.7 (0.9-7.2)	-0.4 (-0.48 to -0.32)
Republic of Kazakhstan	462.9 (98.6-954.7)	3.6 (0.8-7.3)	763 (171.7-1551.4)	4.2 (0.9-8.6)	0.29 (0.03 to 0.55)
Republic of Kenya	383.3 (88.5-831.9)	5 (1.2-11)	1745.1 (410.3-3690.3)	8.1 (2-16.8)	1.77 (1.68 to 1.85)
Republic of Kiribati	7.1 (2-14.1)	19.1 (5.3-37)	16.8 (4.2-34.9)	23.6 (6-48.5)	0.7 (0.6 to 0.79)
Republic of Korea	927.2 (218.4-1806.4)	3.7 (0.9-7.3)	2120.3 (475.4-4130.3)	2.3 (0.5-4.5)	-1.42 (-1.58 to -1.25)
Republic of Latvia	66.7 (15.6-141)	1.9 (0.4-4.1)	119.8 (27.1-246.5)	3.1 (0.7-6.3)	1.5 (1.35 to 1.65)
Republic of Liberia	135.9 (31.7-265.8)	11.6 (2.9-23.2)	317.1 (79.5-670.2)	12.1 (3.1-24.7)	0.37 (0.08 to 0.67)
Republic of Lithuania	79.4 (19.6-166)	1.8 (0.4-3.8)	149.2 (36.8-312.4)	2.5 (0.6-5.4)	0.84 (0.72 to 0.96)
Republic of Madagascar	231.5 (50.2-471.3)	5 (1.1-10.3)	506.5 (110.3-1068)	5.1 (1.1-10.8)	0.06 (-0.02 to 0.13)
Republic of Malawi	198.6 (42.4-404.6)	5.7 (1.2-11.2)	517.3 (117.8-1042.7)	7.5 (1.8-15)	0.69 (0.5 to 0.88)
Republic of Maldives	14 (3.5-26.9)	15.7 (4-30.4)	22 (5.4-43.1)	6.3 (1.6-12.1)	-3.3 (-3.52 to -3.07)
Republic of Mali	366.2 (83.2-736.4)	10 (2.4-19.7)	707.3 (161.2-1441.5)	8.1 (1.9-16.2)	-0.6 (-0.69 to -0.51)
Republic of Malta	20.2 (5-39.9)	5 (1.2-9.9)	37.9 (9.4-74.8)	3.8 (0.9-7.5)	-0.75 (-1.08 to -0.42)
Republic of Mauritius	100.6 (24-195.6)	13.5 (3.3-26.2)	532.9 (134.7-1008.7)	29.1 (7.4-55.7)	3.05 (2.58 to 3.52)
Republic of Moldova	91.5 (20.7-191.9)	2.2 (0.5-4.6)	155.5 (36.9-328.4)	2.7 (0.6-5.7)	0.46 (0.26 to 0.67)
Republic of Mozambique	243.6 (51.8-503.5)	4.6 (1-9.4)	605.7 (148.4-1238.7)	5.9 (1.4-12.2)	1.19 (1.06 to 1.33)
Republic of Namibia	54.2 (12-110.2)	8.4 (1.9-17.2)	117.8 (28-249)	8.2 (2-17.1)	-0.39 (-0.65 to -0.12)
Republic of Nauru	0.8 (0.2-1.6)	17.5 (3.9-33.7)	1.7 (0.4-3.1)	28.6 (7.4-54.5)	1.55 (1.43 to 1.66)
Republic of Nicaragua	123.3 (31.4-261.9)	7.6 (2-15.9)	631.4 (148.1-1273)	12.1 (2.9-24)	1.97 (1.66 to 2.28)
Republic of Niue	0.4 (0.1-0.7)	16.6 (4.1-31.4)	0.7 (0.2-1.4)	31.9 (7.2-66.8)	2.27 (2.2 to 2.34)
Republic of Palau	1.7 (0.4-3)	17.2 (4.4-30.4)	5.7 (1.4-11)	27.2 (6.5-53)	1.76 (1.65 to 1.88)
Republic of Panama	88.3 (20.9-174.4)	5.8 (1.4-11.4)	365.6 (85.9-720.5)	8.2 (1.9-16.2)	1.19 (0.87 to 1.52)
Republic of Paraguay	218.6 (52.2-412.2)	9.8 (2.4-18.6)	586.6 (152.6-1116.8)	9.8 (2.5-18.4)	0.17 (0.03 to 0.32)
Republic of Peru	1228.8 (287-2408.3)	10.6 (2.5-20.8)	3570.1 (901.6-7033.7)	10.6 (2.7-20.8)	-0.26 (-0.47 to -0.06)
Republic of Poland	2861.8 (699.6-5566.1)	6.7 (1.7-13)	2304.4 (582.2-4536.8)	3.3 (0.8-6.6)	-2.59 (-2.96 to -2.22)
Republic of Rwanda	160.8 (35.3-325.3)	6.3 (1.5-12.6)	262.5 (57.9-530.8)	4.7 (1.1-9.6)	-1.82 (-2.18 to -1.46)
Republic of San Marino	1.2 (0.3-2.3)	3.3 (0.8-6.5)	2.1 (0.6-4.1)	2.5 (0.7-4.9)	-0.28 (-0.48 to -0.07)
Republic of Senegal	432.8 (103-862.4)	13.6 (3.3-27.1)	874.5 (207.7-1854.6)	10.9 (2.7-22.7)	-1.06 (-1.24 to -0.88)
Republic of Serbia	489.5 (107.8-1020.8)	4.8 (1.1-9.8)	593.3 (146.3-1208.8)	3.7 (0.9-7.5)	-0.8 (-0.99 to -0.62)
Republic of Seychelles	6.1 (1.5-11.6)	10.9 (2.6-20.6)	14.7 (3.8-28.7)	12.5 (3.2-24.7)	0.54 (0.39 to 0.69)
Republic of Sierra Leone	137.8 (33.7-262)	7.1 (1.7-13.2)	233.3 (51.7-476.1)	6.1 (1.4-12)	-0.52 (-0.6 to -0.44)
Republic of Singapore	97.1 (25.3-186.9)	4.7 (1.2-9)	295.6 (72.5-571.3)	3.5 (0.9-6.7)	0.61 (0.11 to 1.11)
Republic of Slovenia	71.3 (18.5-144.7)	2.9 (0.8-6)	118.8 (31-228.6)	2.6 (0.7-5.1)	-0.19 (-0.33 to -0.04)
Republic of South Africa	1116.9 (255-2196.7)	5.1 (1.2-10)	3365.2 (811.1-6890.2)	7 (1.7-14.2)	1.23 (1.01 to 1.45)
Republic of South Sudan	197.3 (42.4-412.9)	8.2 (1.8-16.8)	396.1 (97.3-815.3)	11.1 (2.9-23.3)	0.88 (0.7 to 1.06)
Republic of Sudan	796.6 (173.5-1727.3)	8.4 (1.9-18)	2352.9 (567.5-4729)	10.4 (2.5-20.5)	0.76 (0.58 to 0.95)
Republic of Suriname	38.6 (9.5-69.6)	14.1 (3.6-25.3)	104.9 (26.9-204.8)	16.4 (4.2-31.9)	0.71 (0.5 to 0.91)
Republic of Tajikistan	54.6 (11.8-117.1)	2 (0.4-4.2)	114.3 (24.5-240.7)	1.8 (0.4-3.8)	-0.43 (-0.56 to -0.3)
Republic of Trinidad and Tobago	76 (19.4-133.1)	9 (2.3-15.7)	313 (80.8-579.2)	16.4 (4.2-30.4)	2.53 (2.21 to 2.85)
Republic of Tunisia	303.2 (70.3-635.8)	6.2 (1.5-12.9)	1011 (242.5-2062)	7.8 (1.9-15.9)	0.82 (0.76 to 0.89)

Republic of Turkey	4113.1 (1022.8-7956.7)	12.4 (3.1-24.1)	9018.1 (2296.5-17912.8)	9.8 (2.5-19.8)	-0.51 (-0.71 to -0.3)
Republic of Uganda	338.8 (73.9-712.6)	5.7 (1.3-11.8)	866.8 (192.8-1794.4)	6.3 (1.5-12.7)	-0.01 (-0.17 to 0.15)
Republic of Uzbekistan	413.7 (86.3-825.9)	3.5 (0.7-6.9)	1575.5 (342.9-3196.4)	5.4 (1.2-11)	0.88 (0.42 to 1.35)
Republic of Vanuatu	10.3 (2.3-22.8)	16.9 (3.8-37.4)	29.7 (6.9-57.2)	16.2 (3.9-30.5)	-0.19 (-0.25 to -0.13)
Republic of Yemen	410.6 (84.6-885.8)	8.7 (1.8-19.3)	917 (213.4-1935.5)	6.4 (1.6-13.4)	-1.2 (-1.34 to -1.06)
Republic of Zambia	230.8 (54.6-472.3)	8.6 (2-16.9)	693.1 (157.9-1457.1)	9.9 (2.3-19.9)	0.05 (-0.13 to 0.23)
Republic of Zimbabwe	333.3 (74.2-667.3)	8.1 (1.9-16.1)	799.7 (192.3-1725.4)	10.2 (2.5-21.8)	0.82 (0.36 to 1.28)
Republic of the Congo	144.7 (35.2-303.1)	13.8 (3.5-28.4)	330.9 (76.5-703.5)	11.6 (2.9-24.5)	-0.78 (-0.91 to -0.64)
Republic of the Gambia	27.1 (6.5-53.8)	8.1 (2-15.6)	93 (20.6-190.5)	9.1 (2.1-18.8)	0.14 (0 to 0.28)
Republic of the Marshall Islands	2.5 (0.5-5.5)	15.1 (3.1-33.2)	10.4 (1.6-30.3)	29.9 (4.5-88.3)	2.47 (2.33 to 2.61)
Republic of the Niger	270.9 (65.5-578.7)	9 (2.2-18.7)	662.4 (153.2-1474.5)	7.3 (1.8-15.7)	-0.75 (-0.82 to -0.69)
Republic of the Philippines	1479.1 (344-2962.3)	5.3 (1.3-10.5)	6110.1 (1454.5-11766.8)	7.2 (1.8-14.2)	1.43 (1.28 to 1.57)
Republic of the Union of Myanmar	1603.1 (403.5-2976.4)	6.8 (1.8-12.5)	2419.2 (566.8-4797.9)	4.9 (1.2-9.7)	-1.18 (-1.24 to -1.12)
Romania	1407.8 (339.1-2735.6)	5.3 (1.3-10.3)	1818.1 (440.7-3440)	5.2 (1.3-9.9)	0.41 (0.07 to 0.76)
Russian Federation	3736.3 (846.8-7890.5)	2.2 (0.5-4.5)	5599.1 (1343.1-11509.7)	2.4 (0.6-4.9)	-0.03 (-0.19 to 0.12)
Saint Kitts and Nevis	6.4 (1.8-11.3)	18.7 (5-33.5)	14 (3.5-25)	20 (4.9-35.8)	1.02 (0.7 to 1.33)
Saint Lucia	11.6 (3-20.6)	13.6 (3.5-24)	35 (8.7-63.2)	14.8 (3.7-26.8)	0.72 (0.47 to 0.97)
Saint Vincent and the Grenadines	7.2 (1.8-12.7)	10.2 (2.6-18.1)	20.2 (5-35.9)	14.7 (3.6-26.2)	1.76 (1.47 to 2.06)
Slovak Republic	286.8 (68.7-590.4)	4.8 (1.1-10)	358.4 (89.4-724.7)	3.9 (1-7.8)	-0.69 (-0.79 to -0.59)
Socialist Republic of Viet Nam	1577.9 (357.4-3019.8)	4.1 (0.9-7.7)	3526.8 (804.9-7062.6)	3.7 (0.8-7.4)	-0.19 (-0.32 to -0.07)
Solomon Islands	23.5 (4.6-48.8)	17.2 (3.5-34.8)	43 (10.1-84.5)	11.4 (2.7-21.8)	-1.55 (-1.77 to -1.34)
State of Eritrea	54.2 (11.4-113.4)	5.2 (1.2-10.8)	144.8 (31.7-299.7)	5.8 (1.3-12)	0.3 (0.22 to 0.38)
State of Israel	345 (82.1-693)	7.5 (1.8-15.2)	605.2 (156.7-1211)	4.6 (1.2-9.3)	-1.35 (-1.69 to -1.01)
State of Kuwait	66.2 (15.9-130.8)	8.4 (2.1-16.3)	180 (45.2-343.1)	5.9 (1.4-11.4)	-0.76 (-0.96 to -0.57)
State of Libya	138.7 (33.3-273.7)	7.1 (1.7-14)	631.7 (158.6-1307.5)	11 (3-22)	2.11 (1.87 to 2.34)
State of Qatar	13.2 (2.9-27.4)	10 (2.3-19.9)	140.9 (32.2-279.7)	17.3 (4-33.3)	1.81 (1.25 to 2.37)
Sultanate of Oman	62.8 (13.9-124)	9.1 (2.1-17.7)	286.3 (69.8-566.5)	12.5 (3.3-24.1)	1.55 (1.26 to 1.85)
Swiss Confederation	295 (72.4-593.9)	2.8 (0.7-5.6)	522.9 (130.6-1022.2)	2.5 (0.6-4.9)	0.17 (-0.05 to 0.4)
Syrian Arab Republic	709.2 (170.9-1396.7)	13.4 (3.3-26.2)	1585.6 (397.6-3248.2)	12.2 (3.1-25.2)	-0.77 (-1.06 to -0.47)
Taiwan (Province of China)	1880.9 (459.5-3420)	13.6 (3.3-24.7)	3911.7 (1041.3-7251.7)	9 (2.4-16.9)	-0.91 (-1.15 to -0.67)
Togolese Republic	90.3 (22.4-180)	8.3 (2.1-16.1)	295.5 (69.8-611.8)	8.6 (2.1-17.3)	-0.06 (-0.14 to 0.01)
Tokelau	0.2 (0-0.3)	12.3 (2.9-25.1)	0.3 (0.1-0.5)	18.3 (4.3-34.4)	1.54 (1.46 to 1.63)
Turkmenistan	91.8 (20.2-183.8)	4.5 (1-9.1)	324.2 (75-655.3)	7.4 (1.7-14.8)	1.6 (1.26 to 1.94)
Tuvalu	1.1 (0.3-2.2)	16.6 (3.8-32)	2.2 (0.5-4.4)	21.3 (5.2-42.2)	0.94 (0.87 to 1)
Ukraine	1019.6 (231-2134.9)	1.5 (0.3-3.2)	1496.3 (360.2-3227.6)	2 (0.5-4.4)	1.18 (0.99 to 1.37)
Union of the Comoros	9.7 (2.3-20)	5.4 (1.3-11.2)	25.5 (5.8-49.9)	5.6 (1.3-11)	-0.09 (-0.21 to 0.03)
United Arab Emirates	62.5 (14.9-123.2)	9 (2.2-17.6)	609.7 (154.7-1167.7)	15 (3.9-28.9)	3.77 (3.14 to 4.39)
United Kingdom of Great Britain and Northern Ireland	1633 (381.3-3286.7)	1.8 (0.4-3.7)	2331.2 (570.9-4579.5)	1.8 (0.4-3.5)	0.24 (0.13 to 0.35)
United Mexican States	4995.7 (1254.2-9422.6)	11.4 (2.9-21.3)	23663 (6121.2-44090.2)	18.1 (4.7-33.7)	2.05 (1.45 to 2.65)
United Republic of Tanzania	639.3 (145.4-1330.4)	6.2 (1.4-12.5)	1890.5 (429.5-3930.2)	7.5 (1.7-15.5)	0.44 (0.39 to 0.5)
United States Virgin Islands	8.5 (2.2-15.7)	9.4 (2.5-17.2)	15.1 (4-29.7)	10 (2.6-19.7)	0.68 (0.51 to 0.85)
United States of America	14892.4 (3770.6-29071.1)	4.7 (1.2-9.2)	51064.7 (13553.8-92511.3)	9.2 (2.5-16.7)	2.34 (2.16 to 2.53)

Table S3 The (p, d, q), AIC, and Ljung-Box (*P*-value) values of the ARIMA model for the CKD burdens attributable to diet low in whole grains in globally.

Measure	Sex	Model(p, d, q)	AIC	BIC	Ljung-Box(*P*-value)
Number					
Deaths					
	Both	(0,2,1)	363.31	366.12	0.6456
	Female	(0,2,1)	328.1	330.9	0.8213
	Male	(0,2,1)	322.47	325.27	0.6341
DALYs					
	Both	(0,2,0)	546.23	547.63	0.4863
	Female	(0,2,0)	504.3	505.71	0.773
	Male	(0,2,1)	510.34	513.14	0.8904
ASR					
Deaths					
	Both	(1,1,0)	-303.25	-298.95	0.9954
	Female	(1,1,0)	-309.23	-304.93	0.8925
	Male	(0,1,1)	-291.37	-287.07	0.9988
DALYs					
	Both	(1,1,0)	-120.12	-117.25	0.9376
	Female	(1,1,0)	-127.82	-123.52	0.3498
	Male	(1,1,0)	-109.48	-106.61	0.8677

DALYs, Disability-adjusted life years; ASR, Age-standardized rate;AIC, Akaike information criterion; BIC, bayesian information criterion; ARIMA, autoregressive integrated moving average.

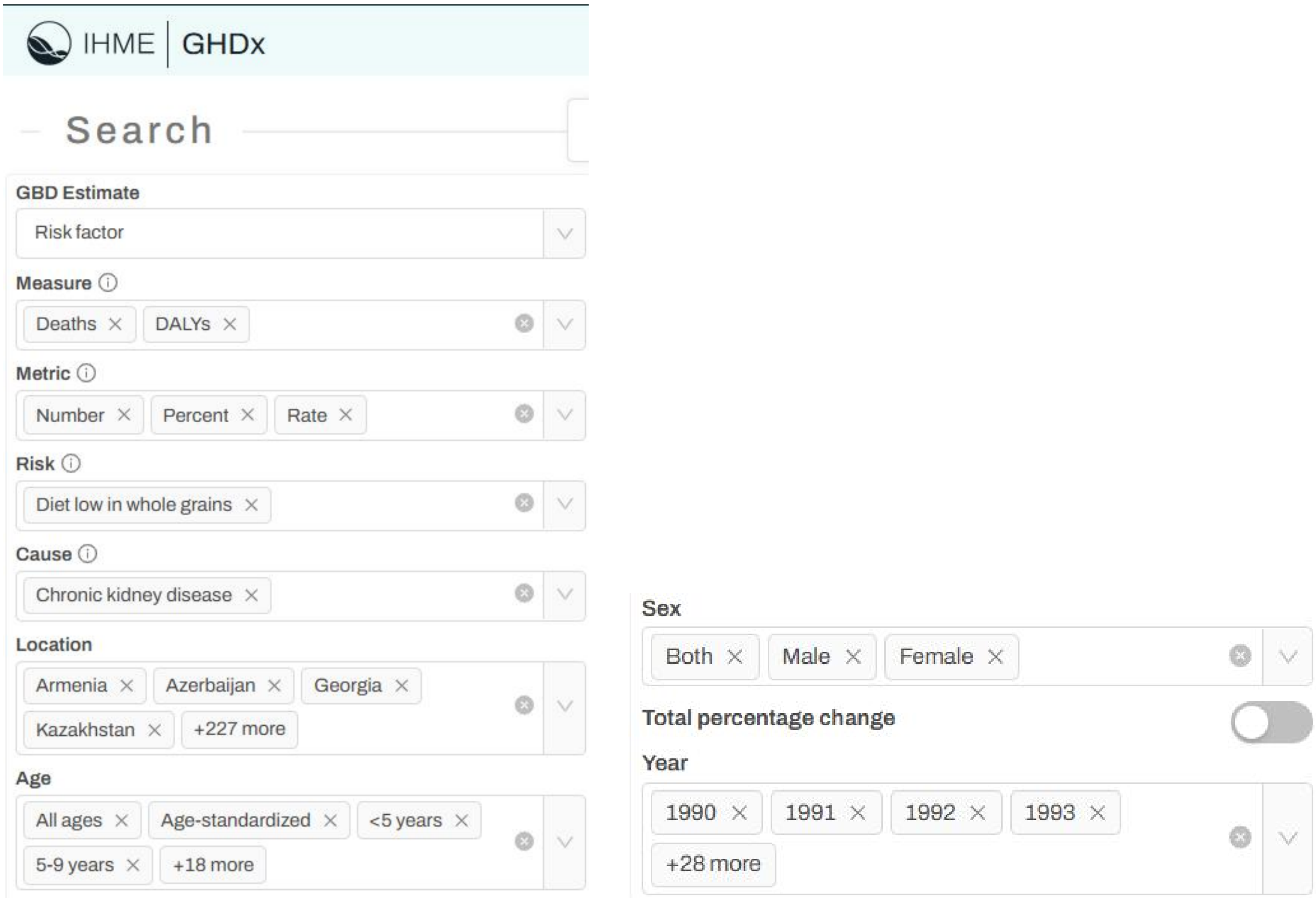

Figure S1 Data Retrieval and Download Steps

A detailed explanation is as follows:

（1）. In the GBD Estimate category, select Risk factor.
（2）. In the Measure category, select Deaths; DALYs.
（3）. In the Metric category, select Number, Percent, Rate.
（4）. In the Risk category, select Diet low in whole grains.
（5）. In the Cause category, select Chronic kidney disease.
（6）. In the Location category, select Global, 5 SDI regions, 21 subregions, and 204 countries and territories.
（7）. In the Age category, select All ages, Age-standardized, and every 5-year age group.
（8）. In the Sex category, select Both, Male, Female.
（9）. In the Year category, select 1990, 1991… 2021.
